# Chemical Bonds Containing Hydrogen: Choices for Hydrogen Carriers and Catalysts

**DOI:** 10.1002/anie.202423661

**Published:** 2025-03-21

**Authors:** James Cashel, Dai Yan, Rui Han, Hyangsoo Jeong, Chang Won Yoon, John Arnold Ambay, Yongfeng Liu, Alison T. Ung, Limei Yang, Zhenguo Huang

**Affiliations:** ^1^ School of Civil and Environmental Engineering University of Technology Sydney Broadway Ultimo New South Wales 2007 Australia; ^2^ Center for Hydrogen and Fuel Cells Korea Institute of Science and Technology 5 Hwarang‐ro 14‐gil Songbuk‐gu Seoul 02792 South Korea; ^3^ Department of Chemical Engineering Pohang University of Science and Technology (POSTECH) Cheongam‐ro, Nam‐gu, Pohang Gyeongbu 37673 South Korea; ^4^ School of Materials Science and Engineering Zhejiang University 38 Zheda Rd, Yuquan Campus Hangzhou 310027 China; ^5^ School of Mathematical and Physical Sciences University of Technology Sydney Broadway Ultimo New South Wales 2007 Australia

**Keywords:** Ammonia, Borohydrides, Formic acid, Hydrogen storage, Liquid organic hydrogen carriers

## Abstract

Compounds containing B─H, C─H, N─H, or O─H bonds with high hydrogen content have been extensively studied as potential hydrogen carriers. Their hydrogen storage performance is largely determined by the nature of these bonds, decomposition pathways, and the properties of the dehydrogenation products. Among these compounds, methanol, cyclohexane, and ammonia stand out due to their low costs and established infrastructure, making them promising hydrogen carriers for large‐scale storage and transport. They offer viable pathways for decarbonizing society by enabling hydrogen to serve as a clean energy source. However, several challenges persist, including the high temperatures required for (de)hydrogenation, slow kinetics, and the reliance on costly catalysts. To address these issues, strategies such as chemical modification and catalyst development are being pursued to improve hydrogen cycling performance. This review highlights recent progress in hydrogen carriers with B─H, C─H, N─H, or O─H bonds. It examines the fundamental characteristics of these bonds and carriers, as well as advances in catalyst development. Our objective is to offer a comprehensive understanding of current state of hydrogen carriers and identify future research directions, such as molecular modification and system optimization. Innovations in these areas are crucial to advance hydrogen storage technologies for a large‐scale hydrogen deployment.

## Graphical abstract

Hydrogen carriers utilizing B─H, C─H, N─H, and O─H bonds are evaluated for their potential in hydrogen storage and release. Key candidates such as methanol, ammonia, and formic acid are examined with a focus on critical factors influencing performance, including the nature of chemical bonds, reaction pathways, and catalytic processes. Both fundamental and applied research efforts are crucial to advancing hydrogen carrier technologies for large‐scale hydrogen deployment.

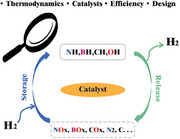

## 1 Introduction

Hydrogen storage is crucial for a hydrogen‐based economy, with both physical and chemical methods being explored. Physical methods such as compression and liquefaction are commonly used but have low hydrogen density and significant energy losses.^[^ ^1^ ^]^ Hydrogen carriers with high hydrogen capacity, such as liquid organic hydrogen carriers (LOHCs) and ammonia, have therefore attracted strong attention. Key performance criteria include hydrogen density, cost, operation temperature and pressure, kinetics of (de)hydrogenation, cycling stability, and environmental impact. From a thermodynamics perspective, a high‐efficiency system should feature hydrogen‐rich compounds with the change in Gibbs free energy (*ΔG*) of around 0 and a slight endothermic (Δ*H* > 0) dehydrogenation, minimizing energy barriers for hydrogen release. Although entropy change (Δ*S*) plays a role, enthalpy change is generally more influential to reaction efficiency.^[^ ^2^ ^]^

Hydrogen‐rich molecules consisting of light elements such as B, N, C, and O are notable for their high hydrogen capacities. Their (de)hydrogenation processes typically involve forming and dissociating the X─H bond (where *X* = B, C, N, and O). Research primarily aims to enhance thermodynamics and kinetics through novel molecule design,^[^ ^3^, ^4^, ^5^, ^6^, ^7^, ^8^ ^]^ new catalysts,^[^ ^9^, ^10^, ^11^, ^12^, ^13^, ^14^, ^15^ ^]^ nanosizing,^[^ ^16^, ^17^, ^18^ ^]^ compositing,^[^ ^19^, ^20^, ^21^, ^22^ ^]^ and blending.^[^ ^23^, ^24^, ^25^, ^26^ ^]^ There have been reviews focusing on specific carriers such as ammonia,^[^ ^27^, ^28^, ^29^ ^]^ alcohols,^[^ ^15^, ^30^, ^31^ ^]^ borohydrides,^[^ ^32^, ^33^, ^34^, ^35^ ^]^ and LOHCs.^[^ ^36^, ^37^, ^38^ ^]^

The carrier's bond type impacts the ease of hydrogen cycling by these carriers. LOHCs are regenerated through direct hydrogenation, while others, like ammonia borane, require multistep chemical processing. The reaction is evaluated using the enthalpy change (Δ*H*), but the onset of any chemical reaction requires surmounting an activation energy barrier (*E*_a_); thus, a lower *E*_a_ suggests more favorable kinetics for the reaction.^[^ ^35^ ^]^ The hydrogen release rate is impacted by the X─H bond type. Bond dissociation energy (BDE) is based on homolytic cleavage, a process in which a chemical bond breaks to form free radicals. Therefore, BDE serves as a useful parameter for initially evaluating or predicting the dehydrogenation characteristics of cyclic LOHCs (C─H) and NH_3_ (N─H). However, certain carriers undergo heterolytic bond cleavage. For instance, formic acid dehydrogenation can proceed via both homolytic (C─H → C* + H^+^) and heterolytic (O─H → O⁻ + H⁺) pathways to produce H₂. Additionally, while BDE can be correlated with reaction enthalpy, this is not always the case, as reaction enthalpy depends on the reactants and products (i.e., it depends on reaction). It is noted that by modifying the molecular structure the BDE can be tuned, leading to variability in performance among carriers with the same X─H bond. For instance, methylcyclohexane (CH_3_C_6_H_11_) and cyclohexane (C_6_H_12_), as well as ammonia borane (NH_3_BH_3_) and lithium aminoborane (LiNH_2_BH_3_), exhibit different performances despite having similar X─H bonds.

For us to have a comprehensive understanding of the impact of X─H bonds and relevant strategies to tune them to improve hydrogen storage performance, this review highlights recent research on hydrogen carriers with B─H, C─H, N─H, or O─H bonds. It starts from the features of these bonds and covers key compounds and catalysts. We also discuss challenges and potential solutions for enhancing hydrogen storage performance. We note that for practical applications, it is essential to consider factors such as cost, melting point, toxicity, vapor pressure, and so on. However, these are out of the scope of this review.

**Figure 1:**
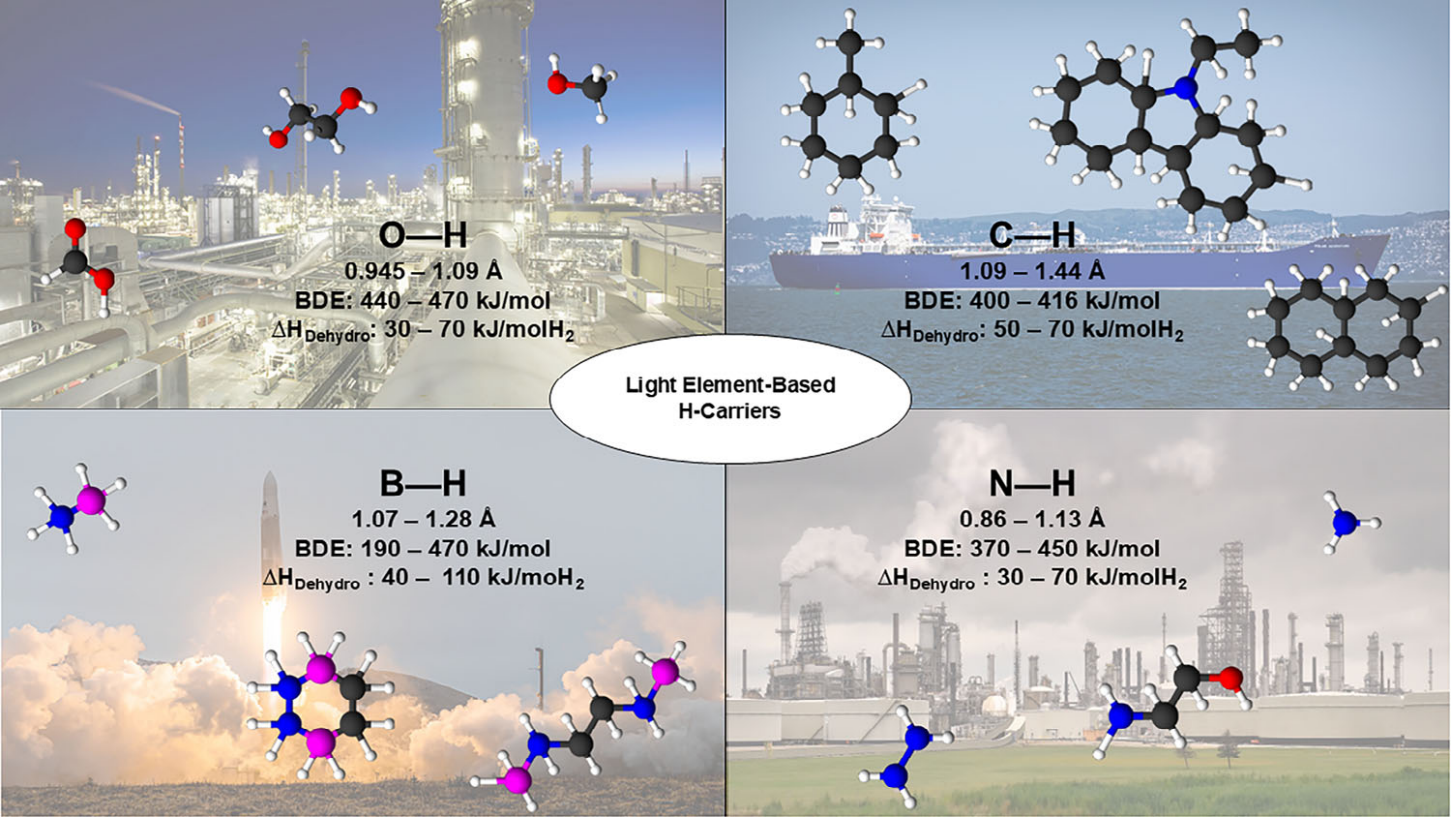
Typical hydrogen carriers are classified based on the X─H bonds. All data were generalised from the CRC Handbook of Chemistry and Physics (95th Edition) and supplementary references. Colour code: Oxygen (red), carbon (black), hydrogen (white), nitrogen (blue), and boron (magenta).^[^ ^39^, ^40^, ^41^ ^]^

As mentioned, BDE is the energy needed to break the X─H bond. BDE can be measured using calorimetry, electrochemistry, and spectrometry, but results can vary, so caution is needed when discussing precise values. Properties like bond length, polarity, and partial charges influence the dehydrogenation enthalpy, affecting hydrogen carrier performance. Compounds with moderate hydrogenation enthalpies (Δ*H *< 0) are preferred since waste heat can be recycled to power dehydrogenation (Δ*H *> 0). Figure 1 summarizes the typical bonds involved, focusing on one type of X─H bond responsible for hydrogen release during cycling such as C─H in LOHCs, N─H in amines, O─H in alcohols, and B─H in borohydrides. Note that blending different X─H types, such as in 2‐ethanolamine, NH_3_BH_3_ and Mg(BH_4_)_2_NH_3_, can significantly alter enthalpy, which will be covered in this review as well. The following sections will outline these bonds in the context of hydrogen storage.

## 2 C─H Bond‐Based Hydrogen Carriers

### 2.1 Choice of Molecules

Activating the carbon‐hydrogen (C─H) bond is a fundamental chemical reaction essential for producing polymers, fine chemicals, and pharmaceuticals.^[^ ^42^, ^43^, ^44^ ^]^ In LOHCs, this activation is crucial for hydrogen storage and release. Typically, thermally induced dehydrogenation with the use of catalysts is employed for C─H carriers; however, this process is thermodynamically uphill due to the high reaction enthalpies ranging from 50 to 70 kJ mol^−1^ H₂.^[^ ^45^, ^46^ ^]^

**Figure 2:**
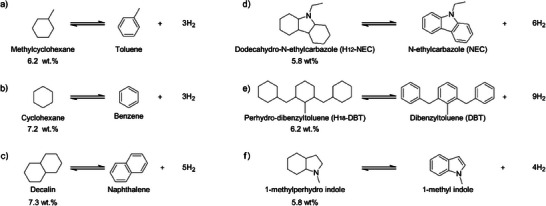
The C─H bond‐based systems for hydrogen storage.

LOHCs have garnered attention for their variety, stability, low cost, and high hydrogen capacity.^[^ ^47^, ^48^, ^49^, ^50^ ^]^ They consist of a hydrogen‐depleted (H_0_‐LOHC) and a hydrogen‐saturated (H_x_‐LOHC) organic pair, enabling hydrogen storage and release through catalytic (de)hydrogenation. Notable pairs include methylcyclohexane (MCH)/toluene (TOL), perhydro‐dibenzyltoluene (H_18_‐DBT)/dibenzyltoluene (H_0_‐DBT), perhydro‐benzyltoluene (H_12_‐BT)/ benzyltoluene (H_0_‐BT), and perhydro‐N‐ethylcarbazole (H_12_‐NEC)/*N*‐ethylcarbazole (NEC). Although linear alkanes have slightly lower BDEs, cycloalkanes offer a more stable backbone, minimizing degradation during repeated (de)hydrogenation.^[^ ^39^, ^51^, ^52^, ^53^ ^]^ The carriers shown in Figure 2 are the most studied LOHCs due to their easy availability. These systems are economically attractive and typically exhibit hydrogen capacities of 6–8 wt.% H (60–62 g H₂/L). However, high dehydrogenation enthalpies (60–70 kJ mol^−1^ H₂) necessitate elevated temperatures, reducing the net energy output. For example, dehydrogenation of H_18_‐DBT consumes almost 30% of the energy of the stored hydrogen.

The MCH/TOL system is one of the earliest and most studied LOHCs.^[^ ^54^, ^55^, ^56^ ^]^ It has a hydrogen storage capacity of 6.2 wt.% H and 47.7 g H_2_/L, and an energy density of 1.55 kWh/L. Dehydrogenation typically requires harsh conditions, with 99% conversion of MCH to TOL demanding ∼300 °C and 1 bar H_2_. Additionally, the low flashpoint of TOL/MCH poses safety concerns, and toluene is an environmental hazard and potential carcinogen.^[^ ^57^, ^58^ ^]^ To address specific issues with the TOL/MCH system, researchers investigated (di)benzyltoluene systems. These carriers have hydrogen capacities around 6.2 wt.% H, moderate volumetric capacities (54–57 g H₂/L), and dehydrogenation enthalpies around 63–65 kJ mol^−1^ H₂).^[^ ^46^ ^]^ Mixtures of (di)benzyltoluene regioisomers are liquids with wide temperature ranges (melting point = ─34 °C; boiling point = 390 °C for H_0_‐DBT), exhibit excellent thermal robustness, low flammability, and have low toxicological profiles.^[^ ^57^, ^58^, ^59^, ^60^, ^61^ ^]^ Importantly, these mixtures are already used in large‐scale industrial applications as heat transfer fluids, ensuring industrial acceptance and availability at low cost. However, the dehydrogenation enthalpy for H_18_‐DBT is 65 kJ mol^−1^ H₂, requiring temperatures above 250 °C.

**Figure 3:**
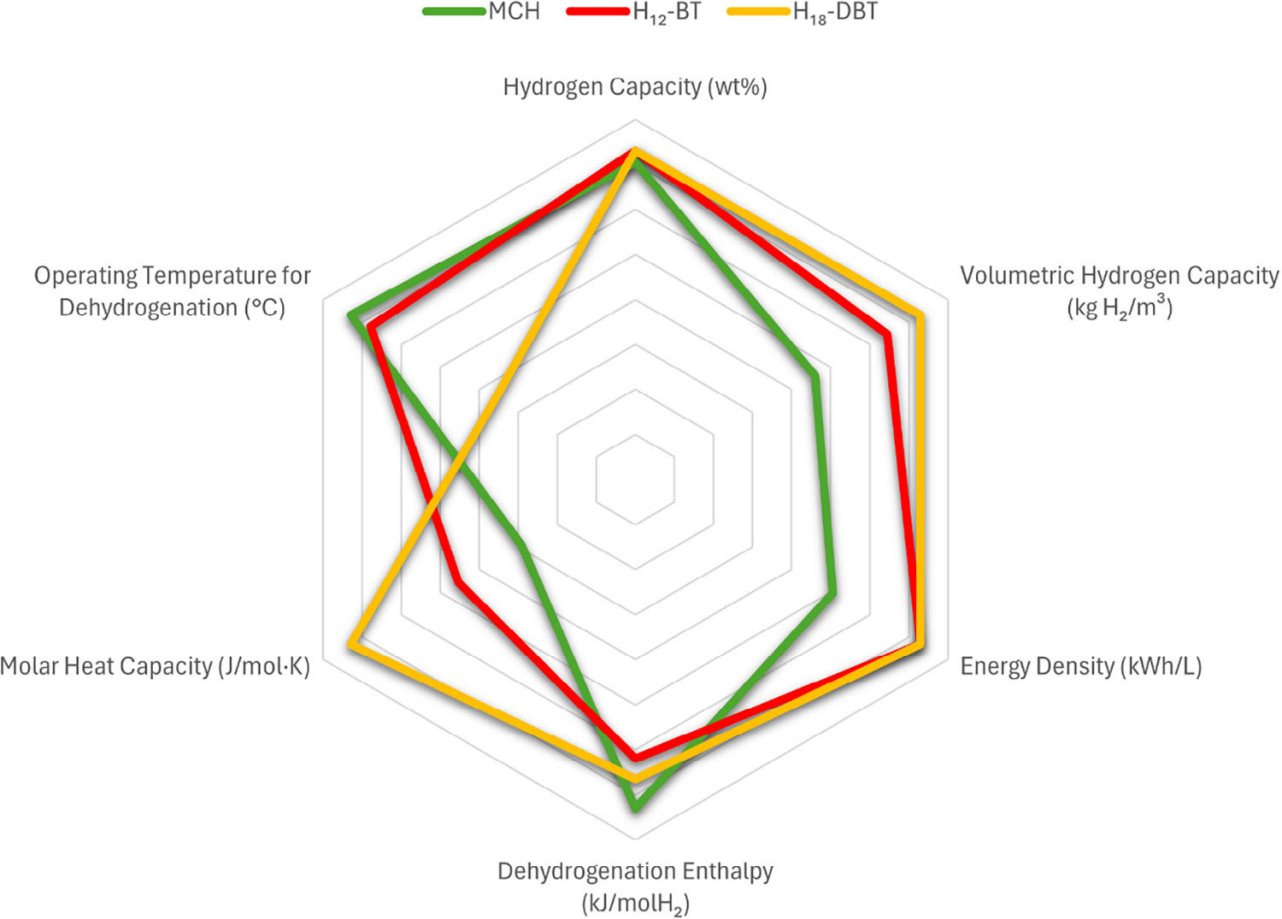
Comparison of key parameters for MCH, H18‐DBT, and H12‐DBT.^[^ ^45^, ^62^, ^63^, ^64^, ^65^ ^]^

Understanding structural influences on hydrogen storage performance can help develop energy efficient solutions for LOHC applications. To explore the impact of molecular structure, we compare MCH/TOL, H_18_‐DBT/H_0_‐DBT, and H_12_‐BT/H_0_‐BT (Figure 3). MCH and H_18_‐DBT share similar BDEs around 416 kJ mol^−1^, but their different sizes lead to varied operating conditions and storage capacities. H_18_‐DBT has a larger framework, facilitating hydrogen release at lower temperatures (280–320 °C) and offers better thermal management with a heat capacity of approximately 450 J/mol·K, compared to MCH's 180 J/mol·K. This increased thermal buffering enhances temperature control and energy efficiency, reducing energy input for hydrogen release.^[^ ^45^, ^62^ ^]^ H_18_‐DBT serves as a thermal reservoir, improving system stability and allowing for waste heat recovery, which can enhance overall energy efficiency.

Additionally, H_18_‐DBT has a volumetric capacity of 57 g H₂/L and an energy density of about 1.8 kWh/L, making it ideal for stationary storage where energy density and thermal management are crucial.^[^ ^63^ ^]^

H_12_‐BT, with a similar hydrogen content around 6.2 wt.% H exhibits good thermal stability within a 290–320 °C range.^[^ ^64^, ^65^ ^]^ This increased cycling stability makes H_12_‐BT more robust over repeated hydrogenation and dehydrogenation. H_12_‐BT offers better cycling stability than H_18_‐DBT because it produces fewer by‐products. An LOHC with more C─C bonds tends to deactivate dehydrogenation catalysts more rapidly under similar reaction conditions. For example, Pt catalysts are typically used for LOHC dehydrogenation, but they cannot only activate the desired C─H bonds but also cleave C─C bonds, ultimately leading to coke formation and catalyst deterioration. H_18_‐DBT creates multiple heavy, high‐boiling by‐products that cause coking, while H_12_‐BT primarily forms methylfluorene and methane. Although methylfluorene has slower adsorption kinetics due to its C5‐ring, its overall accumulation is much lower than the coke precursors from H_18_‐DBT.^[^ ^66^ ^]^ Methylfluorene was previously considered an LOHC and operates similarly to H_12_‐BT, however, its isomers have high melting points and induce coke formation significantly, so its fast desorption is necessary to enhance cycle stability.^[^ ^64^, ^65^ ^]^

Distribution of H_12_‐BT is easier due to its lower viscosity. Compared to a viscosity of 49 mPa·s for H_18_‐DBT, pumping H_12_‐BT is more feasible at 7 mPa·s. This is beneficial for storage and pumping of the material in colder regions.^[^ ^64^ ^]^ H_12_‐BT also has a lower molar heat capacity of 375 J/mol·K.^[^ ^45^ ^]^ This means that H_12_‐BT requires less energy for temperature increases but may have greater temperature fluctuations during hydrogenation. Regarding their dehydrogenation enthalpies, H_12_‐BT has a lower dehydrogenation enthalpy of around 63 kJ mol^−1^ H₂ compared to H_18_‐DBT's 65 kJ mol^−1^ H₂, potentially reducing the overall energy input for hydrogen release. However, H_18_‐DBT has a higher volumetric hydrogen capacity (57 g H₂/L) versus H_12_‐BT (54 g H₂/L), making it more suitable for energy dense applications.^[^ ^46^ ^]^ These trade‐offs highlight how subtle adjustments in molecular structure can affect efficiency and durability in practical settings.

Heteroatom substitution, especially with nitrogen, is an effective strategy for reducing energy barriers. A notable nitrogen‐containing system, H_12_‐NEC/H_0_‐NEC, can store 5.8 wt.%H, with a volumetric density of nearly 70 g H₂/L and an energy density of 2.5 kWh/L (Figure 2d).^[^ ^63^ ^]^ Its dehydrogenation enthalpy is about 50 kJ mol^−1^ H₂, much lower than pure hydrocarbon LOHCs such as MCH. In H₁₂‐NEC, the BDE of the vicinal C─H bond is 340 kJ mol^−1^, compared to 400 kJ mol^−1^ for perhydrofluorene (the carbonaceous analogue).^[^ ^39^, ^40^, ^41^ ^]^ This decrease stems from nitrogen's influence on electron distribution in the aromatic system, where nitrogen's lone pair electrons stabilize radical intermediates formed during dehydrogenation, facilitating homolytic cleavage of the C─H bond.^[^ ^67^ ^]^ Although the reduction is confined to carbons next to nitrogen, its presence effectively lowers dehydrogenation enthalpy. For instance, the dehydrogenation of piperidine to pyridine requires 63 kJ mol^−1^ H₂, while cyclohexane to benzene requires 69 kJ mol^−1^ H₂, highlighting nitrogen's role in stabilizing reaction states.^[^ ^41^ ^]^ However, H_0_‐NEC has a melting point around 70 °C, which poses challenges for its practical operation. Researchers found that eutectic mixtures of *N*‐alkyl‐substituted carbazoles can lower melting point to 24 °C.^[^ ^37^, ^68^ ^]^ However, nitrogen decreases stability, making these compounds, including their hydrogenated forms, more prone to dealkylation and ring‐opening reactions, which results in degradation and capacity loss.^[^ ^49^, ^69^ ^]^ This reduced thermal stability over repeated cycles raises concerns about durability and hydrogen purity. Balancing improved dehydrogenation thermodynamics with stability is crucial when designing nitrogen‐containing LOHCs.^[^ ^49^, ^69^ ^]^ Indoles are another nitrogen‐containing LOHC that benefit from heteroatom substitution. Vostrikov et al. studied their kinetics and thermodynamics, revealing that hydrogenating indene (−99.1 kJ mol^−1^) is more exothermic than indole (−37.9 kJ mol^−1^) due to strain release in the five‐membered ring.^[^ ^70^ ^]^ Li et al. reported that the 2‐methyl indole (5.76 wt.% H) system was dehydrogenated using a 5 wt.% Pd/Al₂O₃ catalyst at 190 °C with near complete conversion in 4 h.^[^ ^71^ ^]^ However, its melting point (around 60 °C) poses challenges as it experiences a phase change during operation, complicating system design, and energy management.^[^ ^71^ ^]^ To address this, Yang et al. studied 1‐methyl indole, which has a lower melting point (−20 °C), making it more suitable for practical applications.^[^ ^72^ ^]^ Complete hydrogenation to 1‐methylperhydro indole was achieved with a 5 wt.% Ru/Al₂O₃ catalyst at 130 °C and 60 bar H_2_, and dehydrogenation achieved 100% selectivity in 5 h (Figure 2f). Table 1 summarizes the bond characteristics and dehydrogenation enthalpy of various LOHCs.

**Table 1:** Physical and thermodynamic properties of the common C─H bond‐based hydrogen carriers at standard conditions (hydrogenated molecules).^[^ ^39^, ^40^, ^41^, ^45^, ^63^, ^68^, ^72^, ^73^ ^]^

	Melting Points (°C)		Boiling Points (°C)					
Chemical	H_2_‐Rich	H_2_‐Lean	H_2_‐Rich	H_2_‐Lean	Bond Length (Å)	BDE (kJ mol^−1^)	Enthalpy (kJ mol^−1^, kJ mol^−1^ H_2_)	Hydrogen Capacity (wt.% H, g H_2_/L)
Cyclohexane	6	5.5	80	80	1.10–1.12	416	205.5, 68.5	7.2, 56
Methylcyclohexane	−126	−95	100	110	1.10–1.12	416	204.9, 68.3	6.2, 47.7
Decalin	−30	80	189	218	1.09–1.11	400	319.5, 63.9	7.3, –
N‐ethyl‐carbazole	−85	70	280	166	1.37–1.44	416	303.6, 50.6	5.8, 69
H_18_‐Dibenzyltoluene	−58	−40	350	390	1.10–1.12	416	588.6, 65.4	6.2, 57
H_12_‐Benzyltoluene	−80	−30	264	277	1.10–1.12	416	381, 63.5	6.2, 54
1‐Methylperhydro indole	25	95	180	238	1.20–1.30	NA	207.6, 51.9	5.8, –

As promising as LOHCs are for hydrogen storage, tackling issues such as cost, energy demands, and environmental impact, and safety is essential. Several techno‐economic assessments have highlighted the economic and logistical advantages of LOHCs over physical hydrogen but also noted the significant energy costs associated with the dehydrogenation process.^[^ ^74^, ^75^, ^76^, ^77^ ^]^ Nevertheless, there have been several commercial demonstrations.^[^ ^78^, ^79^, ^80^, ^81^ ^]^ Chiyoda corporation successfully demonstrated using MCH/TOL to distribute 100 tons of hydrogen over 10 months.^[^ ^80^, ^82^, ^83^ ^]^ As these large‐scale developments progress, the need to proactively address environmental and safety issues becomes imminent. Many LOHCs have been extensively studied, providing a solid understanding of their physical and chemical hazards.^[^ ^36^, ^47^, ^57^, ^58^, ^84^, ^85^ ^]^ Most LOHCs have acceptable biodegradation; however, they are considered potentially toxic carcinogens, so preventing environmental spill and leak must be prioritized in the LOHC space.^[^ ^57^ ^]^

### 2.2 Catalysts: Established and Emerging Choices

C─H bond formation and cleavage typically require noble metals due to the high enthalpy,^[^ ^73^, ^86^ ^]^ and Pt‐ or Ru‐based catalysts have been studied extensively. Although homogeneous catalysts offer higher activity rates, they are challenging to recycle due to being in the same phase as the carriers. As a result, our review emphasizes heterogeneous catalysts as they offer more practical use cases. Recent developments in hydrogenation and dehydrogenation for LOHCs are summarized in Tables 2 and 3.

**Table 2:** Summary of catalysts for the hydrogenation of LOHCs.

Reactant	Catalyst	Temperature (°C)	Pressure (bar H_2_)	Loading	TOF (h^−1^)	Notes	Ref.
Toluene	3 wt.% Ru/NHPC	100	14	1 h, 25 mg_CAT_, and 94.4 mmol_TOL_	15 792	–	[87]
	Pd‐Ni/SiO_2_ (co‐SEA)	150	70	6 h, 0.15g_CAT,_ and 25 mL_TOL_	11 520	–	[88]
	2.5 wt.% Ru‐OMC‐0.22	150	40	85 mg_CAT_, and 30 mL_TOL_	16 884	–	[89]
H_0_‐DBT	0.5 wt.% Ru/MgO	170	50	150 min, 0.1 mol%_CAT_, and 15 g_H0‐DBT_	3840	–	[90]
	Rh/Al_2_O_3_	150	45	0.25 mol%_CAT_, and 300 g_H0‐DBT_	–	70% conversion (10 h)	[91]
H_0_‐NEC	1.3 wt.% Ru/YH_3_	130	70	3 h, 5 wt.%_CAT_, and 1 g_H0‐NEC_	–	Turnover Number = 319 h^−1^	[92]
	RhCo/y‐Al_2_O_3_	90	60	8 h, 0.5 g_CAT_, and 5 g_HO‐NEC_	–	50% conversion (2 h), 100% conversion (8 h)	[93]
Benzene	1.25 wt.% Ru/(Ni/Ni(OH)_2_)‐15.57 wt.% Ni/C	60	53	1 h, 50 mg_CAT, and_10 mL_BENZ_	13 522	–	[94]
	0.024 wt.% Ru‐1 wt.% Ni/C	60	48	2 h, 50 mg_CAT_, and 10 mL_BENZ_	474 300	–	[95]
	1.25 wt.% Ru‐1.40 wt.% Co/C	60	53	0.2 h, 50 mg_CAT_, and 10 mL_BENZ_	91 052	–	[96]

**Table 3:** Summary of catalyst for the dehydrogenation of LOHCs.

Reactant	Catalyst	Temperature (°C)	Loading	TOF (h^−1^)	Notes	E_a_ (kJ mol^−1^)	Ref.
Methylcyclohexane	Pt/Ce_14_‐Mg‐Al‐O	350	10 h, 0.5 g_CAT_, and 0.1 mL_MCH_ min^−1^ (Fixed‐bed reactor)	–	1358.6 mmolH_2_ gPt^−1^ min^−1^	–	[97]
	Pt/Al_2_O_3_	240	20 h, 30 mg_CAT_ 6 uL_MCH_ min^−1^	12 900	100% selectivity & >50% conversion (100% conversion at 300 °C)	–	[98]
	Pt/Al_2_O_3_	300	50 mg_CAT_, 30 uL_MCH_ min^−1^	50.4	656 mmolH_2_ gPt^−1^ min^−1^, 100% selectivity	38.05	[99]
H_18_‐DBT	Pt/Al_2_O_3_–P–Hac	320	80 min, 5 g_CAT_, and 20 mL_H18‐DBT_		Degree of dehydrogenation = 96%	205	[100]
	Pt/Al_2_O_3_–P–Hac	270	0.3 mol%_CAT,_ 0.037 mol_H18‐DBT_	15 480	–	–	[60]
	Pt/Al_2_O_3_	300	100 min, 0.05 mol%_CAT_	16 200	–	–	[60]
H_12_‐NEC	Pd_1.2_Cu/rGO	180	7 h, 0.1 g_CAT_, and 1.5 g_H12‐NEC_		100% selectivity	–	[101]
	Pd/C	200*	0.05 mol_H12‐NEC_, n_CAT_:n_H12‐NEC_ = 1:1000		0.829 gH_2_ gPd^−1^ min^−1^	69.4	[102]
	Pd/C	180	1 h, 0.06 g_CAT_	692	90.5% conversion, 35.5% selectivity for H_0_‐NEC	–	[103]
Cyclohexane	2Cu/SBA‐15	350	150 mg_CAT_	5578	<25% conversion, 100% selectivity	46.5	[104]
	Pt/TiAl_2_	400	>100 h, 2 g_CAT_, and 0.2 mL_CHEX_ min^−1^	1711	93.2% conversion	–	[105]
	Pt/CN_1.9_	210	90 min, 120 mg_CAT_, and 420 uL_CHEX_	211.1	96% conversion	36.2	[106]

Platinum remains a preferred (de)hydrogenation catalyst due to its strong ability to activate C─H bonds, and controlling particle size and surface acidity are crucial to avoid unwanted C─C rupture.^[^ ^14^ ^]^ Optimizing dehydrogenation catalysts is critical for hydrogen storage and release, as hydrogenation reactions are generally more favorable. For example, Pt particle sizes of 2.3–2.7 nm is optimal for H_12_‐BT or H_18_‐DBT dehydrogenation.^[^ ^107^ ^]^ Notably, Chiyoda corporation has developed a Pt‐based potassium‐doped catalyst with highly uniform pore sizes, enabling continuous hydrogen discharge from MCH for thousands of hours.^[^ ^108^ ^]^ Single‐atom catalysts (SACs) are a breakthrough technology that can transform how we conduct chemical reactions. Recent studies have demonstrated that SACs can enhance reaction efficiency and lower operational expenses by minimizing metal loading.^[^ ^109^, ^110^ ^]^ A Pt/CeO_2_ SAC demonstrated TOFs for dehydrogenation of almost 30000 mol_H2_ mol Pt^−1^ h^−1^ at 350 °C and approached 800 mol_TOL_ mol Pt^−1^ h^−1^ for hydrogenation (120 °C).^[^ ^10^ ^]^ Transition metal‐based catalysts have also been explored. Toluene was fully hydrogenated within 2 h at 200 °C under 20 bar H_2_ facilitated by a mixture of Co, Ni, and Mo oxides on zeolite.^[^ ^111^ ^]^ Metal hydrides have drawn attention for LOHC hydrogenation, particularly under higher pressures and temperatures. For DBT, several metal hydrides (Mg_2_NiH_4_, MgH_2_, and LaH_3_) were explored, with Mg_2_NiH_4_ performing the best.^[^ ^112^ ^]^ Under 60 bar H_2_ at 280 °C, Mg_2_NiH_4_ converted 48% of DBT in 8 h and 77% in 20 h.

**Figure 4:**
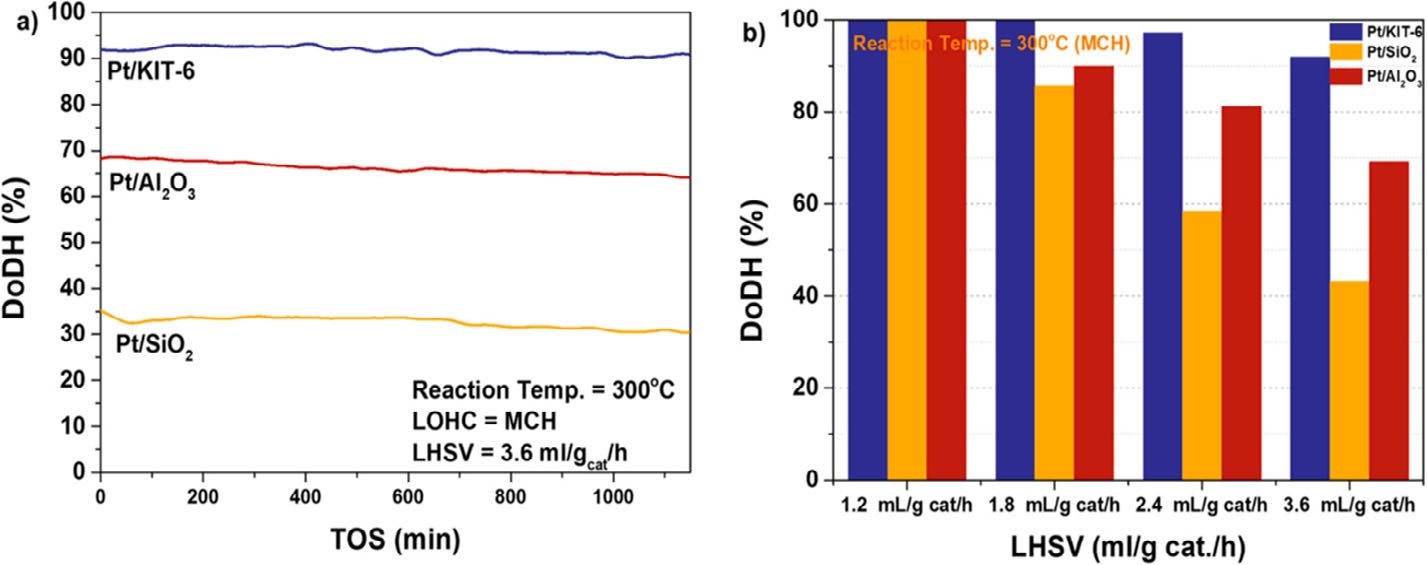
Catalytic performance of Pt/Al_2_O_3_, Pt/SiO_2_, and Pt/KIT‐6 catalysts for MCH dehydrogenation: a) Stability testing, b) reaction at varying LHSV values.^[^ ^115^ ^]^

To improve the catalytic activity of LOHC (de)hydrogenation, various promoters (e.g., S, K, Sn, Mn, and Cu) and support materials such as KIT‐6, TiO_2_, and CeO_2_ have been explored.^[^ ^10^, ^106^, ^113^, ^114^, ^115^, ^116^, ^117^ ^]^ The active metal particles are typically dispersed on a porous support, such as silica, alumina, or carbon, to increase the metal surface area. Recently, sulfur modified catalysts are known to be highly active for H_12_‐BT dehydrogenation.^[^ ^86^, ^118^, ^119^, ^120^ ^]^ Molybdenum carbide (Mo_2_C) was employed as a promoter for the Pt/Al_2_O_3_ catalyzed hydrogenation of DBT.^[^ ^121^ ^]^ In less than 6 h under 14 bar H_2_ at 160 °C, about 80% of H_0_‐DBT was reformed into H_18_–DBT. Mesoporous silica supported Pt (Pt/KIT‐6) demonstrated better catalytic activity than Pt on disordered SiO_2_ or Al_2_O_3_ (Figure 4).^[^ ^115^ ^]^ This improved performance can be attributed to higher surface area, pore volume, and better dispersion. Hierarchically porous carbons (HPCs) have been studied, as their unique porous structures help reduce resistance and shorten diffusion pathways, improving mass transfer. Tang et al. investigated nitrogen‐doped HPCs supported Ru (Ru/NHPC) for the hydrogenation of toluene.^[^ ^87^ ^]^ Their study found that the Ru/NHPC system achieved the highest conversion rate (72%) and selectivity for MCH (98%), with a turnover frequency (TOF) of 15792 h^−1^. Other supports had lower activity, with Ru/HPC converting 38% and Ru/AC only 8%. This was attributed to the enhanced metal–support interaction, which increased mass transfer, hydrophilicity, and electron density at the active sites. Additionally, Ru/NHPC had higher adsorption energy, suggesting a lower reaction barrier and, consequently, higher activity for toluene hydrogenation.

Thermal dehydrogenation is effective but has major drawbacks, including the need for high temperatures (typically over 200 °C) and high pressures due to its endothermic reaction. Additionally, catalyst deactivation and degradation occur over time.^[^ ^122^ ^]^ To address challenges in LOHC dehydrogenation, different methods have been investigated to improve catalyst stability and reduce energy input. Through photocatalysis, Zhang et al. demonstrated a platinum (Pt) catalyst on black titanium dioxide (TiO₂) with an activation energy of about 9 kJ mol⁻¹ for room‐temperature cyclohexane dehydrogenation.^[^ ^123^ ^]^ Visible light can promote iridium(III)‐catalyzed hydrogen production from *N*‐heterocycles.^[^ ^124^ ^]^ Microwave‐assisted dehydrogenation is also highly effective, where site‐selective heating of carbon‐supported Pt improves hydrogen purity and release during MCH dehydrogenation, with the catalyst maintaining performance over 67 000 cycles.^[^ ^122^ ^]^ Despite these advances, challenges remain, such as the high cost of noble metals and scalability issues. Readers can explore recent reviews on photocatalytic and microwave‐assisted nonoxidative processes for more insights.^[^ ^125^, ^126^ ^]^

Beyond microwave‐assisted and photocatalytic strategies, progress to improve long‐term stability can be achieved through optimizing reaction conditions, innovating reactor designs, and refining the reaction composition. The C─C bond is more thermally stable than the C─N bond, giving hydrocarbon‐based structures an advantage over those with heteroatoms. This thermal stability is crucial for long‐term applications.^[^ ^66^, ^127^ ^]^ Nitrogen‐containing LOHCs show promise for hydrogen release but struggle with material compatibility, thermal stability, high melting points, viscosity, and limited availability. This highlights the need for advancements in hydrocarbon systems.

Long‐term stability also relies on preserving catalyst activity and reducing deactivation from coking or poisoning. For instance, treating H_12_‐BT catalysts with hydrogen and purifying feedstock with molecular sieves extended stability.^[^ ^65^ ^]^ Techniques such as reactive distillation that combines reaction and separation improve durability by reducing fouling and preserving catalyst efficiency over multiple cycles.^[^ ^66^ ^]^ This reactor type enables H_12_‐BT dehydrogenation at temperatures as low as 200 °C under reduced pressure. This is achieved by lowering the hydrogen partial pressure, which shifts the thermodynamic equilibrium toward dehydrogenation. In contrast, conventional fixed‐bed reactors operating at atmospheric pressure require temperatures of 290–300 °C for the same reaction. The ability to reduce the reaction temperature offers two key advantages: it decreases the energy required for dehydrogenation and minimizes the formation of side products, such as the cyclization by‐product methylfluorene. Experimental designs, such as fixed‐bed and pellet string reactors help effectively assess catalyst deactivation under realistic conditions, ensuring the long‐term reliability of hydrogen storage materials.^[^ ^65^, ^127^ ^]^

### 2.3 Summary and Conclusions for C─H Bonds

LOHCs are recognized for their thermal and chemical stability, high hydrogen capacity, and durable C─C backbone, which minimizes degradation during (de)hydrogenation processes. Most LOHCs have relatively low toxicity and established safety protocols for handling. They are also cost‐effective and widely available, which is conducive to large‐scale deployment.

However, LOHCs have limitations, including the high energy needed for dehydrogenation, and its slow kinetics. Expensive platinum‐based catalysts are the main choice which increase the operation costs. For future application, it is crucial to develop low‐cost catalysts that still resist deactivation from coking and poisoning to ensure long‐term stability. New reactor design should be developed to improve mass and heat transfer, thus accelerating (de)hydrogenation rates. New LOHCs composed of novel molecules or eutectic mixtures should be investigated as well.

## 3 O─H Bond‐Based Hydrogen Carriers

### 3.1 Choice of Molecules

The activation of O─H bonds is critical in various chemical reactions.^[^ ^128^, ^129^, ^130^ ^]^ O─H bond containing compounds studied for hydrogen storage normally contain one or two hydroxyl groups. The O─H bonds are typically shorter than C─H bonds (by 0.1 to 0.2 Å) and require more energy to activate than C─H bonds (BDEs around 440 kJ mol^−1^), but their dehydrogenation enthalpies are in the range of 16–65 kJ mol^−1^ H_2_.^[^ ^14^, ^25^, ^39^, ^131^ ^]^ Compared with C─H bond‐based carriers, O─H carriers dehydrogenate through mechanisms beyond simple homolytic bond cleavage, which will be discussed below.

**Figure 5:**
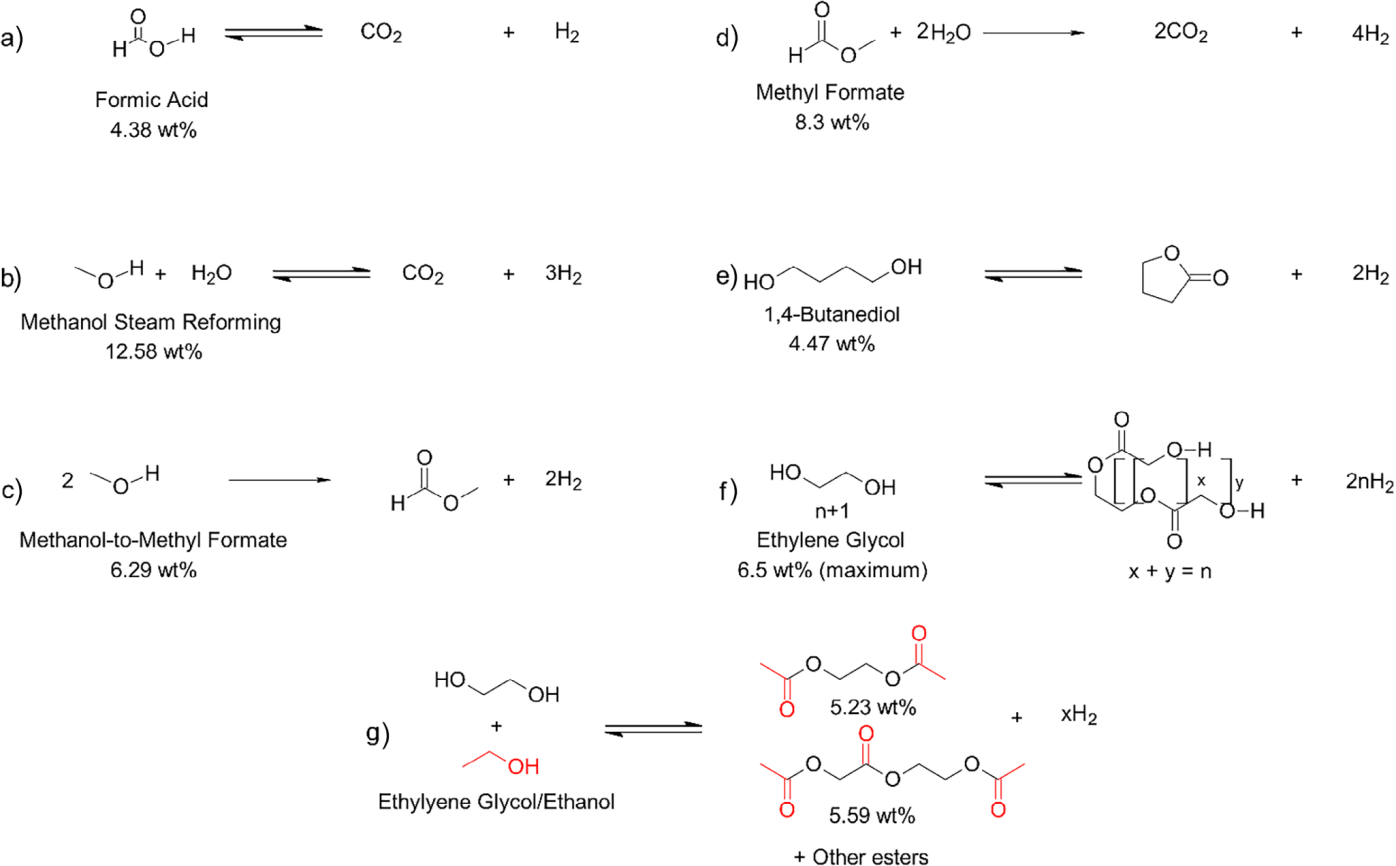
The O─H bond‐based systems for hydrogen storage.

Table 4 and Figure 5 illustrate the O─H bond‐containing compounds under consideration, including methanol, formic acid, and other alcohols. Among these, methanol and formic acid stand out for their ease of handling, reasonable hydrogen densities, and the simple dehydrogenation products (CO_2_/CO/H_2_). Formic acid, with a gravimetric density of 4.4 wt.%H and a volumetric density of 53 g H_2_ L^−1^,^[^ ^132^, ^133^ ^]^ offers an energy density of 1.8 kWh L^−1^.^[^ ^134^, ^135^ ^]^ It surpasses the typical 1.4 kWh L^−1^ of hydrogen tanks used in fuel cell vehicles such as Toyota Mirai.^[^ ^136^ ^]^ Methanol, the simplest alcohol, presents an even more promising profile, with hydrogen densities of 12.6 wt.% H and 98.8 g H_2_ L^−1^, translating to an energy density of 3.26 kWh L^−1^.^[^ ^136^ ^]^ However, we have to examine the energy required to extract hydrogen and reform these molecules. Additionally, the nature of the dehydrogenation products influences the applications of each carrier.

**Table 4:** Physical and thermodynamic properties of the common O─H bond‐based hydrogen carriers under standard conditions (* = properties of CO_2_, # = ethyl acetate, + = based on liquid‐phase dehydrogenation).^[^ ^14^, ^25^, ^39^, ^131^, ^132^, ^133^, ^134^, ^135^, ^136^, ^137^, ^138^, ^139^ ^]^

	Melting Points (°C)		Boiling Points (°C)					
Chemical	H_2_‐Rich	H_2_‐Lean	H_2_‐Rich	H_2_‐Lean	Bond Length (Å)	BDE (kJ mol^−1^)	Enthalpy (kJ mol^−1^, kJ mol^−1^ H_2_)	Hydrogen Capacity (wt.%H, g H_2_ L^−1^)
Formic acid	8.4	−78^*^	100.8	−57*	0.972	469	31.2, 31.2	4.4, 53
Methanol	−97.6	−78^*^	64.7	−57*	0.945	440	131, 43.7^+^	12.6, 98.8
1,4‐Butanediol	20.1	−43.53	235	204	1.09	–	86.4,43.2	4.47, 47
1,2‐Ethandiol	−12.9	–	198	–	0.946–0.947	–	–	6.5
Ethanol	−114	−84^#^	78	77^#^	0.971	441	–	4.3, 35

**CO_2_‐evolving decomposition**:

(1) $${\text{HCOOH}}_{\left(l\right)}⇋{\text{CO}}_{2\left(g\right)}+H_{2\left(g\right)}\left(\Delta H=+31.2\;\text{kJ}\;{\text{mol}}^{-1}\right)$$

(2) $$\begin{array}{cc} & {\text{CH}}_{3}{\text{OH}}_{\left(l\right)}+H_{2}O_{\left(l\right)}⇋{\text{CO}}_{2\left(g\right)}\\ & \;+3H_{2\left(g\right)}\left(\Delta H=+131.0\;\text{kJ}\;{\text{mol}}^{-1}\right) \end{array}$$

(3) $$\begin{array}{cc} & {\text{CH}}_{3}{\text{OH}}_{\left(g\right)}+H_{2}O_{\left(g\right)}⇋{\text{CO}}_{2\left(g\right)}\\ & \;+3H_{2\left(g\right)}\left(\Delta H=+49.2\;\text{kJ}\;{\text{mol}}^{-1}\right) \end{array}$$

(4) $$\begin{array}{cc} & {\text{CH}}_{3}{\text{OH}}_{\left(g\right)}+0.5O_{2\left(g\right)}⇋{\text{CO}}_{2\left(g\right)}\\ & \;+2H_{2\left(g\right)}\left(\Delta H=-192\;\text{kJ}\;{\text{mol}}^{-1}\right) \end{array}$$

There are two primary pathways for accessing the hydrogen stored in formic acid and methanol: (1) CO₂‐evolving decomposition (Equations (1), (2), (3), (4)) and (2) CO‐evolving decomposition (Equations (5), (6), (7)).^[^ ^140^, ^141^, ^142^, ^143^ ^]^ However, these pathways are not equally accessible to both carriers. CO₂‐evolving pathways are preferred due to their carbon‐neutral potential and their avoidance of CO, which can poison catalysts. Formic acid, with its carboxyl group (−COOH), can undergo a decarboxylation reaction, which is thermodynamically favoured (Δ*G*° = −33 kJ mol^−1^) and involves the direct release of CO₂ (Equation 1).^[^ ^137^ ^]^ In contrast, methanol lacks such a functional group, precluding decarboxylation and instead necessitating more complex and energy‐demanding decomposition routes such as steam‐reforming (Equations (2), (3)), partial oxidation (Equation 4) to release hydrogen.^[^ ^30^, ^144^, ^145^, ^146^ ^]^ The activation energy shows the different energy requirements. For formic acid decarboxylation without a catalyst, the barrier was reported to be between 273 and 284 kJ mol^−1^.^[^ ^147^, ^148^ ^]^ Similarly, methanol decomposition in the gas phase without a catalyst has an activation energy barrier up to 334 kJ mol^−1^.^[^ ^149^ ^]^ Thus, methanol's inability to follow a CO₂‐evolving pathway without significant energy input means it cannot use the same thermodynamic efficiency that formic acid enjoys.

Therefore, the thermodynamic behaviors of formic acid and methanol cannot simply be traced to their different structures. Formic acid, despite having a stronger O─H bond (0.972 Å, BDE 470 kJ mol^−1^) compared to methanol (0.945 Å, BDE 440 kJ mol^−1^), requires less energy for dehydrogenation—around 30 kJ mol^−1^, compared to a range of 50 to 130 kJ mol^−1^, depending on methanol's various pathways.^[^ ^39^, ^137^ ^]^

**CO‐evolving decomposition**:

(5) $${\text{HCOOH}}_{\left(l\right)}⇋H_{2}O_{\left(l\right)}+{\text{CO}}_{\left(g\right)}\left(\Delta H=+28.7\;\text{kJ}\;{\text{mol}}^{-1}\right)$$

(6) $${\text{CH}}_{3}{\text{OH}}_{\left(l\right)}⇋{\text{CO}}_{\left(g\right)}+2H_{2\left(g\right)}\left(\Delta H=+128.7\;\text{kJ}\;{\text{mol}}^{-1}\right)$$

(7) $${\text{CH}}_{3}{\text{OH}}_{\left(g\right)}⇋{\text{CO}}_{\left(g\right)}+2H_{2\left(g\right)}\left(\Delta H=+90.2\;\text{kJ}\;{\text{mol}}^{-1}\right)$$

Another critical factor in the application of these carriers is the risk of catalyst poisoning in CO‐evolving pathways (Equations (5), (6), (7)), particularly in methanol decomposition. In comparison, methanol exhibits a higher hydrogen density but requires higher energy to produce hydrogen and may cause catalyst poisoning, while formic acid can release hydrogen with a lower energy demand.

**Formate anion dehydrogenation pathway**:

(8) $${\text{HCOOH}}_{\left(\mathrm{aq}\right)}⇋{\text{HCOO}}_{\left(\mathrm{aq}\right)}^{-}+H^{+}$$

(9)

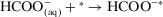

(10) $${{\text{HCOO}}_{\left(g\right)}^{-}}^{*}\to H^{-*}+{\text{CO}}_{2\left(g\right)}$$

(11) $$H^{-*}+H^{+*}\to H^{*}+H^{*}$$

(12) $$H^{*}+H^{*}\to H_{2\left(g\right)}+2^{*}$$

As mentioned, unlike LOHCs, the hydrogen in these decomposition reactions is not released through a simple X─H bond cleavage. Therefore, the energy profile of these systems cannot be explained by BDE alone. This is because the BDE of O─H bonds come from homolytic bond cleavage; however, many O─H carriers start dehydrogenation via heterolytic O─H bond cleavage (e.g., HCOO–H → HCOO^−^ + H^+^), then C─H bond cleavage, followed by combination of the hydride and proton to produce H_2_. Some pathways may proceed through deprotonation, depending on the catalyst (e.g., CH_3_OH → CH_3_O^−^ + H^+^ → H_2_). This is one of the key differences between O─H carriers and C─H carriers. For example, aqueous formic acid decomposition can proceed via the formate anion (HCOO^−^). The formate anion pathway (Equations (8), (9), (10), (11), (12)) proceeds first with the heterolytic cleavage of formic acid, and subsequent adsorption onto the active site (*), where it decomposes into a hydride and CO_2_. The next step involves the adsorption of protons from solution, followed by the formation of H_2_.

**Formic acid dehydrogenation pathway**:

(13)

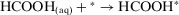



(14)

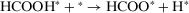

(15) $${\text{HCOO}}^{*}\to {\text{CO}}_{2\left(g\right)}+H^{*}$$

(16) $$H^{*}+H^{*}\to H_{2\left(g\right)}+2^{*}$$

The alternative pathway, formic acid dehydrogenation (Equations (13), (14), (15), (16)), proceeds first with the adsorption of formic acid to the active site, where homolytic cleavage of the O─H bond yields formate. The adsorbed formate then undergoes C─H bond cleavage to yield CO_2_, and the adsorbed hydrogens are combined and desorbed to produce H_2_.

Therefore, the energy of these pathways is not decided by BDE. Kim et al. investigated these mechanisms using a Pd/C catalyst, reporting that the formate anion pathway has a lower activation barrier than the direct dehydrogenation route.^[^ ^150^ ^]^ Specifically, the formic acid dehydrogenation pathway needs to overcome a barrier of around 65 kJ mol^−1^ for O─H cleavage (Equation 14) and around 80 kJ mol^−1^ each for the subsequent C─H cleavage (Equation 15). By comparison, the formate anion pathway has a lower activation energy of −27 kJ mol^−1^ for C─H bond cleavage (Equation 8).

A lower pKa allows formic acid to dissociate easily, generating formate ions (HCOO^−^) that readily coordinate with catalysts to drive dehydrogenation. This coordination step is crucial, as observed in iridium catalysts, where formate ion formation is the rate‐determining step.^[^ ^151^ ^]^ According to Guan et al., pure formic acid would not proceed via dehydrogenation.^[^ ^152^ ^]^ Notably, Kar et al. developed a ruthenium 9H–acridine pincer complex with excellent solubility in formic acid, yielding a CO‐free H_2_/CO_2_ gas stream at room temperature and maintaining stability over a month, achieving a total turnover number of 1 701 150.^[^ ^133^ ^]^

**The formate‐bicarbonate cycle**:

(17) $$\begin{array}{cc} & \text{HCOO}M_{\left(\mathrm{aq}\right)}+H_{2}O_{\left(l\right)}⇋{\text{HCO}}_{3}M_{\left(\mathrm{aq}\right)}+H_{2\left(g\right)}\\ & \left(M=K,\text{Na, and}\;{\text{NH}}_{4}\right) \end{array}$$

**Figure 6:**
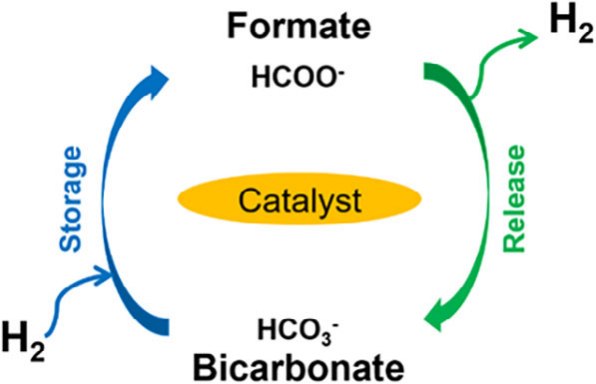
The formate–bicarbonate cycle.

An intriguing alternative is the formate‐bicarbonate system, which offers a CO_2_‐ and CO‐free route to hydrogen production, releasing only H_2_ gas.^[^ ^153^, ^154^, ^155^ ^]^ This system was first noted in 1985 by Zaidman et al. and later demonstrated by Wiener et al.,^[^ ^156^, ^157^ ^]^ and has been further explored with both heterogeneous^[^ ^158^, ^159^, ^160^, ^161^ ^]^ and homogenous catalysts.^[^ ^162^, ^163^, ^164^, ^165^, ^166^, ^167^, ^168^ ^]^ Formate salts are nontoxic solids that can be easily transported and converted into highly concentrated aqueous solutions. Although their hydrogen energy densities are low (1.2–1.6 wt.%H, 20 – 28 gH_2_/L), the simplicity and reversibility of the formate‐bicarbonate equilibrium make them an attractive hydrogen carrier. Small shifts in reaction conditions, facilitated by the low free energy (Δ*G*° = 0 around 50 °C), enable efficient cycling near ambient temperatures–ideal for fuel cell applications.^[^ ^153^, ^154^ ^]^ The cycle for the system follows the formate salt's decomposition into hydrogen and bicarbonates, which subsequently gets regenerated back into the formate salt (Equation 17, Figure 6).

Russo et al. analyzed the thermodynamics and kinetics of the formate–bicarbonate system, showing that the dehydrogenation step is endothermic (Δ*H* = 19.4–49.5 kJ mol^−1^) and favorable at temperatures above 50 °C, while hydrogenation becomes favorable at lower temperatures.^[^ ^154^ ^]^ The hydrogenation of bicarbonate is much more thermodynamically efficient than CO_2_ hydrogenation, with Δ*G*° ≈ 0 at 50 °C, compared to the endergonic Δ*G*° = 33 kJ mol^−1^ for CO_2_ to formic acid. Two approaches exist for this system: (1) hydrogenation under constant pressure for complete conversion and (2) pressurizing the solution to equilibrium, though the latter limits hydrogen yield due to pressure build‐up.^[^ ^154^ ^]^ In practical applications, the stability and cyclability of the catalyst, often Pd or Pt on activated carbon, are essential considerations.^[^ ^153^ ^]^

Papp et al. demonstrated sodium formate with up to 90% regeneration over multiple charge/discharge cycles.^[^ ^169^ ^]^ Using a [RuCl_2_‐(mtppms)_2_]_2_] complex (mtppms = sodium diphenylphosphino benzene‐3‐sulfonate), hydrogenation to sodium formate was achieved with 90% conversion at around 80 °C under 100 bar H_2_ within 200 min. This process was repeated multiple times over 2 days, and only 40–50% of the hydrogen capacity could be achieved. Nakajima et al. explored a Pd‐Au/AC catalyst for the ammonium bicarbonate/formate (HCO_3_NH_4_/HCO_2_NH_4_) system (1.87 wt.%H, 21.2 g H_2_/L).^[^ ^161^ ^]^ They observed that increasing the Au/Pd ratio to 10:1 enhanced bicarbonate hydrogenation and achieved a TOF of 5820 h^−1^, while a 1:1 ratio optimized dehydrogenation with a TOF at 4200 h^−1^. The 3 nm particle size and electron‐poor Au within the alloy improved nucleophilic hydrogen addition onto Pd, enhancing catalytic performance. Bi et al. introduced a rechargeable hydrogen battery using Pd nanoparticles on reduced graphene oxide (PdNP/rGO). This system efficiently cycled HCO_3_K – HCO_2_K under mild conditions (80–100 °C, 40 bar H_2_).^[^ ^170^ ^]^ Although studies advance specific catalysis and hydrogen storage aspects, challenges remain across efficiency, stability, and scalability.

Methanol can be converted to hydrogen under relatively mild conditions (150–300 °C) compared to other fuels such as methane (800–1000 °C) or ethanol (400–1000 °C).^[^ ^171^, ^172^ ^]^ Also, methanol decomposition avoids the issue of by‐products such as ether, ketal, or other oxidized by‐products faced by ethanol (methane, diethyl ether, and acetaldehyde).^[^ ^171^ ^]^ Methanol decomposes into CO and H_2_ through an endothermic process (90, 45 kJ mol^−1^ H_2_) that can occur catalytically or thermally at high temperatures. At 650 to 750 °C, methanol breaks down further into formaldehyde, which decomposes into CO and H_2_.^[^ ^173^ ^]^ Methanol steam reforming (MSR, Figure 5b) is enthalpically favorable (49.7, 16.6 kJ mol^−1^ H_2_) and has been industrially adopted.^[^ ^145^ ^]^ MSR typically operates above 200 °C,^[^ ^144^ ^]^ but can proceed near room temperature using dehydrogenation enzymes and iridium complex catalysts.^[^ ^174^ ^]^ However, MSR produces CO in varying concentrations (1%–8%), which can be toxic and cause catalyst deactivation.^[^ ^144^ ^]^ Industrially, the water–gas shift reaction mitigates CO formation by converting it to CO_2_ and H_2_.^[^ ^175^ ^]^

An alternative hydrogen production route involves partially dehydrogenating methanol to methyl formate (MF). Liquid below 32 °C, MF offers advantages as a nontoxic, noncorrosive hydrogen carrier (Figure 5c).^[^ ^24^, ^176^ ^]^ Research on methanol‐MF systems began in the 1990s,^[^ ^26^ ^]^ and MF has since been recognized for its favorable hydrogen density (8.4 wt.%H), and its low free energy (Δ*G*° = −16.6 kJ mol^−1^) suggests a low barrier to recycling.^[^ ^177^ ^]^ Recently, Sang et al. demonstrated catalytic MF dehydrogenation with the formation of CO_2_ using a commercial Ru‐pincer complex, achieving a TOF of 44 000 h^−1^ and a TON of 100 000 with no CO detected (Figure 5d).^[^ ^177^ ^]^ Although under‐researched as a hydrogen carrier, MF has industrial relevance, with over 6 Mt produced annually.^[^ ^178^ ^]^

**Ethanol dehydrogenation**:

(18) $$C_{2}H_{5}\text{OH}\to {\text{CH}}_{3}\text{CHO}+H_{2\left(g\right)}\left(\Delta H=+68.7\;\text{kJ}\;{\text{mol}}^{-1}\right)$$

(19) $$\begin{array}{cc} & {\text{CH}}_{3}{\text{CH}}_{2}\text{OH}+{\text{CH}}_{3}\text{CHO}\to {\text{CH}}_{3}{\text{COOCH}}_{2}{\text{CH}}_{3}\\ & \;+H_{2\left(g\right)}\left(\Delta H=-43.45\;\text{kJ}\;{\text{mol}}^{-1}\right) \end{array}$$

(20) $$\begin{array}{cc} & 2{\text{CH}}_{3}{\text{CH}}_{2}\text{OH}\to {\text{CH}}_{3}{\text{COOCH}}_{2}{\text{CH}}_{3}\\ & \;+2H_{2\left(g\right)}\left(\Delta H=+25.25\;\text{kJ}\;{\text{mol}}^{-1}\right) \end{array}$$

**Ethanol reformation**:

(21) $$\begin{array}{cc} & 2{\text{CO}}_{2\left(g\right)}+6H_{2\left(g\right)}\to C_{2}H_{5}{\text{OH}}_{\left(g\right)}\\ & \;+3H_{2}O_{\left(g\right)}\left(\Delta H=-173.7\;\text{kJ}\;{\text{mol}}^{-1}\right) \end{array}$$

Ethanol has also been explored for hydrogen storage. It offers a good capacity of 35 gH_2_/L,^[^ ^179^ ^]^ but the hydrogen production presents challenges with selectivity, often yielding ketones, ethers, acetic acid, and ethylene.^[^ ^14^, ^180^, ^181^, ^182^, ^183^, ^184^ ^]^ Ethanol dehydrogenation initially produces acetaldehyde and hydrogen (Equation 18), followed by a reaction with acetaldehyde to yield ethyl acetate and further hydrogen (Equation 19). Combined, these reactions have an enthalpy of 25.25 kJ mol^−1^ (Equation 20).^[^ ^25^ ^]^ The reformation of ethanol is shown in Equation 21.^[^ ^185^ ^]^ Among all catalysts for hydrogen evolution, Cu‐based ones exhibit the highest selectivity to ethyl acetate, while Ni and Pd are more selective toward methane and carbon monoxide.^[^ ^186^ ^]^

**Ethanol steam reforming**:

(22) $$\begin{array}{cc} & C_{2}H_{5}{\text{OH}}_{\left(g\right)}+3H_{2}O_{\left(g\right)}\to 2{\text{CO}}_{2\left(g\right)}\\ & \;+6H_{2\left(g\right)}\left(\Delta H=+173.5\;\text{kJ}\;{\text{mol}}^{-1}\right) \end{array}$$

Like methanol, ethanol can also undergo steam reforming and partial oxidation under similar conditions.^[^ ^187^, ^188^ ^]^ Ethanol steam reforming (ESR) is endothermic (173.5 kJ mol^−1^), requiring high temperatures (300–600 °C) to proceed efficiently (Equation 22).^[^ ^189^ ^]^ However, side reactions—such as dehydration to acetone, coking, and acetaldehyde decomposition—complicate ESR.^[^ ^190^, ^191^, ^192^, ^193^, ^194^, ^195^, ^196^, ^197^ ^]^ The conversion efficiency depends heavily on the catalysts. Ni‐based catalysts are found to enhance hydrogen production through effective C─C bond scission.^[^ ^189^, ^198^ ^]^ Alumina is a common catalyst support due to its high surface area and stability, but its acidity promotes coke formation. This issue can be mitigated by doping alumina with basic oxides like La_2_O_3_ and ZrO_2_ to neutralize acidic sites and reduce coking.

CO_2_ sorbents such as CaO‐based materials have been employed to enhance hydrogen purity in ESR processes, minimizing the need for energy‐intensive processes such as pressure swing adsorption (PSA). These materials exhibit high carbonation kinetics at 650 °C, synergizing well with Ni catalysts. CaOS‐C5 spheres, for example, led to > 95% hydrogen purity across multiple cycles.^[^ ^199^ ^]^ A 10 wt.% NiO/CaO catalyst tested between 600 and 750 °C produced a gas mixture containing up to 22.3 wt.% H_2_, and CO_2_ absorption declined at higher temperatures.^[^ ^200^ ^]^ Other studies reported absorption capacities ranging from 5.06 to 16.22 mmol CO_2_ g_CAT_^−1^, depending on catalyst composition and synthesis techniques.^[^ ^201^, ^202^, ^203^, ^204^ ^]^ Jo et al. achieved higher absorption rates using a sol–gel synthesized catalyst, with high surface area, leading to absorptions of 15.49 mmol CO_2_ g_CAT_^−1^ at 600 °C and 16.22 mmol CO_2_ g_CAT_^−1^ at 700 °C.^[^ ^203^ ^]^ Di Giuliano et al. reported 8.20 mmol CO_2_ g_CAT_^−1^ at 650 °C using a 3% Ni/CaO‐mayenite catalyst.^[^ ^204^ ^]^

Carriers with multiple O─H groups are less desirable because dehydrogenation competes with dehydration reactions, reducing hydrogen capacity. For instance, glycerol, a by‐product of biodiesel production and key feedstock for high‐value chemicals, has proven challenging to dehydrogenate beyond intermediates such as dihydroxyacetone or lactic acid.^[^ ^205^, ^206^, ^207^ ^]^ Diol compounds, such as 1,4‐butanediol, have also been explored.^[^ ^139^ ^]^ Onoda et al. developed a system using 1,4‐butanediol/ɣ‐butyrolactone with an iridium‐6,6‐dionato‐2,2′‐bipyridine catalyst, achieving a gravimetric hydrogen storage density of 4.47 wt.%H (Figure 5e).^[^ ^208^ ^]^ The reaction yields 96% butyrolactone and hydrogen without solvents, and the same catalyst can facilitate reversible hydrogenation of ɣ–butyrolactone to 1,4–butanediol under 8 bar H_2_ at 130 °C. The Milstein group introduced a reversible liquid hydrogen carrier system using ethylene glycol, with a hydrogen storage capacity of 6.5 wt.%H (Figure 5f).^[^ ^209^ ^]^ Dehydrogenation produces liquid oligoesters, while hydrogenation is achievable using the same ruthenium complex‐based catalyst in a toluene and 1,2‐dimethoxyethane mixture under 40 bar H_2_. Further studies by Milstein and coworkers demonstrated that a combination of ethylene glycol and ethanol could serve as a reversible liquid hydrogen carrier (Figure 5g), offering a hydrogen storage capacity exceeding 5 wt.%H.^[^ ^23^ ^]^ Notably, loading hydrogen occurs at low pressure (5 bar), enhancing its appeal as a LOHC.

The electrochemical decomposition of O─H bond carriers, such as formic acid and methanol, is another effective way to produce high‐purity hydrogen under mild conditions. For instance, Kilic et al. produced 99.999% pure hydrogen at ambient temperature from formic acid.^[^ ^210^ ^]^ Platinum is the best catalyst for electrochemical decomposition due to its high activity and efficiency, but it is prone to fouling from CO intermediates when oxidizing formic acid and methanol.^[^ ^211^, ^212^, ^213^ ^]^ Recent studies aim to develop cheaper electrocatalysts with improved CO tolerance and stability by adjusting binding energies and optimizing surface structures.^[^ ^211^, ^212^ ^]^

### 3.2 Catalysts: Established and Emerging Choices

**Figure 7:**
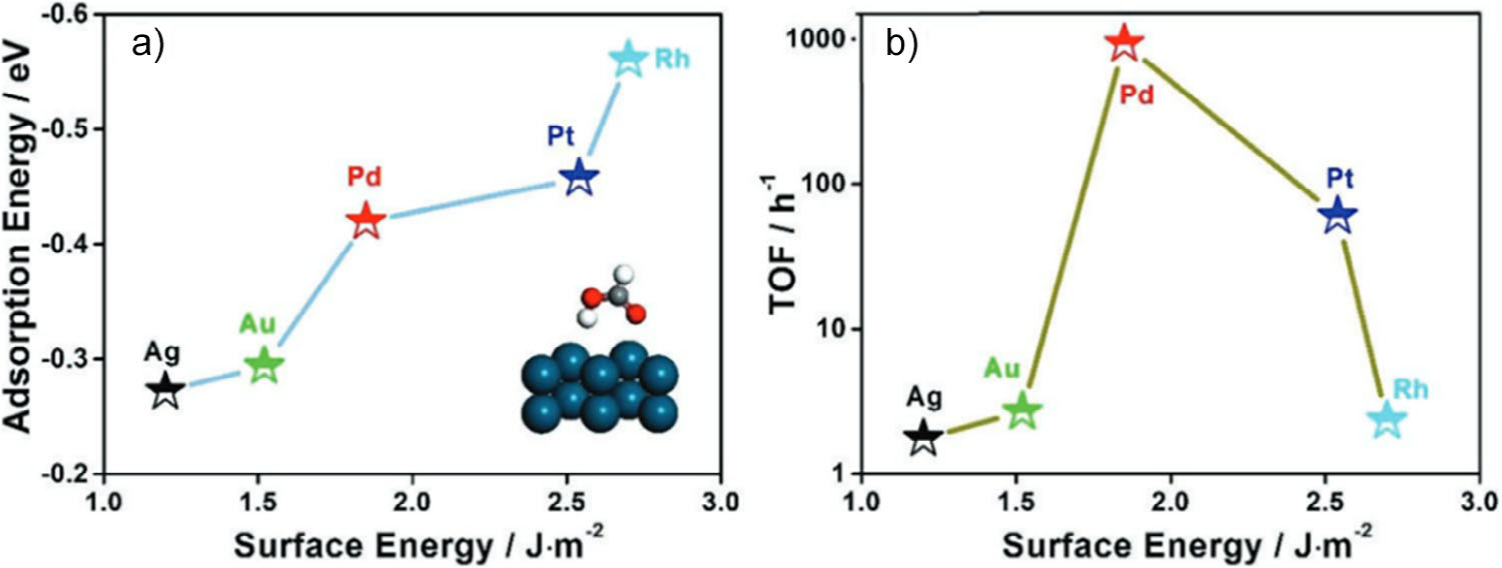
Surface energy, adsorption energy, and activity of noble metals for HCOOH adsorption and decomposition. Inset diagram of A: HCOOH adsorption onto (111) metal surface. Spheres: green‐ metal, white‐H, grey‐C, and red‐O atoms.^[^ ^217^ ^]^

Catalysts play a crucial role in the (de)hydrogenation of O─H bond‐based carriers, especially in key molecules such as formic acid and methanol. For formic acid, the formation of a formate intermediate (HCOO^−^) is essential to facilitate hydrogen release and prevent dehydration pathways that form CO, a known poison to fuel cell catalysts.^[^ ^214^ ^]^ Similarly, catalysts for methanol dehydrogenation must aim for mild conditions while suppressing CO formation. Homogenous catalysts are favored for their high efficiency and mild conditions, allowing precise reaction control. For instance, Wang et al. reported an iridium‐based catalyst achieving TOFs up to 38 236 h^−1^ at 100 °C. Onishi et al. achieved 81 900 h^−1^ in 8 M formic acid at 60 °C.^[^ ^215^ ^]^ Despite their high performance, recycling or recovering homogeneous catalysts remains an engineering hurdle. For more details on FA dehydrogenation, refer to reviews by Onishi et al.^[^ ^216^ ^]^ and Guan et al.^[^ ^152^ ^]^ Heterogeneous catalysts, particularly Pd‐based, while limited to surface activity, are advantageous due to their ease of recovery and longer lifespans. Numerous studies show that Pd nanoparticles delivered superior TOFs compared to non‐Pd alternatives, as shown in Table 5. Effective heterogeneous catalysts must strongly adsorb FA on the metal surface to capture formate and cleavage the C─H bond. Li et al. found a positive correlation between adsorption energy and catalyst surface energy among noble metals, with Pd showing the best efficiency due to its balanced surface energy (Figure 7).^[^ ^217^ ^]^ A suitable promoter can substantially enhance activity, as demonstrated by the sevenfold difference in TOF between PdMn@S‐1 (6860 h⁻¹) and Pd@S‐1 (944 h⁻¹), with Mn promoting charge transfer to Pd to boost its electron density.^[^ ^218^ ^]^ For further insights, consider reviews by Beller,^[^ ^219^ ^]^ Kapteijn,^[^ ^220^ ^]^ Olah,^[^ ^221^ ^]^ and Jiang.^[^ ^222^ ^]^

**Table 5:** List of formic acid dehydrogenation catalysts. (A‐M‐β‐CD = aminopropyltriethoxysilane monochlortriazinyl β‐cyclodextrin, SF = Sodium formate, FA = Formic acid, MPG = Mesoporous graphite, APTES = (3‐Aminopropyl)triethoxysilane).

Catalyst	Support	Temp (°C)	Solution	TOF (h^−1^)	E_a_ (kJ mol^−1^)	Ref.
Co_6_Ag_0.1_Pd_0.9_/RGO	Reduced graphene oxide	25	FA/SF = 2/5	453	43.1	[223]
		40		1674		
		50		2739		
		60		4711		
PdMn@S‐1	S‐1 zeolite	80	2 M FA	6860	56.5	[218]
		60		3338		
		25		610		
Pd@S‐1		80		944		
Mn@S‐1		80		0		
Pd‐WOx/NPCC	N‐doped porous carbon cage	50	1.25 M FA/3.75 M SF	1378	35.9	[224]
Pd/(P)NPCC	Phosphate‐modified N‐doped porous carbon cage			1963		
Pd‐WOx/(P)NPCC				6135		
Cr_0.4_Pd_0.6_/M‐β‐CD‐A	M‐β‐CD‐A	50	1 M FA	5771	49.4	[225]
	APTES			3524		
	M‐β‐CD			168		
	–			61		
	C			376		
	TiO_2_			175		
	ZrO_2_			173		
	SiO_2_			127		
	CeO_2_			102		
	Al_2_O_3_			95		
Au_0.3_Pd_0.7_/A‐M‐β‐CD	A‐M‐β‐CD	50	1 M FA	7352	39.5	[226]
	APTES			3032		
	C			218		
	M‐β‐CD			91		
	–			10		
0.01‐PANI‐Pd/C	C core	30	1.1 M FA/0.8 M SF	5654	24.4	[227]
0.04‐PANI‐Pd/C				4873		
0.1‐PANI‐Pd/C				2073		
0.7‐PANI‐Pd/C				692		
Pd/C	C			2925		
Ni_0.2_Co_0.2_Pd_0.6_‐CeO_x_/NPG	N‐P‐rGO	30	1.0 M FA	6506.8	17.7	[228]
	N‐P‐rGO			3303.4		
	N‐rGO			2308.9		
	P‐rGO			49.8		
	rGO			33		
	–			21.5		
PdAgCr/NH_2_‐MSC	NH_2_‐mesoporous C	75	10 mL 1 M 9:1 FA:SF	6898	22.3	[229]

The catalytic reduction of CO_2_ into FA is crucial for sustainable hydrogen systems. Bases like bicarbonate or carbonate salts often facilitate this process more efficiently than gaseous CO_2_ itself.^[^ ^214^, ^230^ ^]^ Ziebart et al. showed that an iron‐based catalyst could reduce sodium bicarbonate to sodium formate at 100 °C under 60 bar H_2_, achieving a 77% yield and a TON of 7546.^[^ ^231^ ^]^ However, lower pressures of 30 bars dropped the yield to 52% with a TON of only 966. Wang et al. developed a nanoporous nickel catalyst that converted various carbonates into formic acid, reaching 92% conversion at 200 °C under 60 bar H_2_ in 2 h while maintaining stable activity across five cycles.^[^ ^232^ ^]^

Industrial methanol production uses CuO/ZnO/Al_2_O_3_‐based catalysts to convert CO/CO_2_ into methanol at 30–60 bar and 200–300 °C.^[^ ^144^ ^]^ Attempts to efficiently dehydrogenate methanol at milder conditions with minimal by‐products has been challenging. High‐cost catalysts such as ceria–supported palladium^[^ ^233^ ^]^ and platinum^[^ ^234^ ^]^ were tested but are impractical for large‐scale use.^[^ ^14^, ^15^, ^30^ ^]^ Suppressing CO formation is essential to enhancing hydrogen purity. Mo et al. reported an Ag/Ce_20_Zn catalyst achieving 92% conversion and 91% hydrogen selectivity with only 6% CO.^[^ ^235^ ^]^ An Au/CuO/ZnO catalyst with 3% Au yielded hydrogen of 98% purity with complete methanol conversion at 275 °C.^[^ ^13^ ^]^ David et al. investigated methanol decomposition mechanisms on Cu(111) and Cu(100) surfaces, noting a reaction pathway involving methoxy elimination of CO.^[^ ^236^ ^]^ They measured an activation barrier of 46 – 55 kJ mol^−1^, significantly lower than 334 kJ mol^−1^ required for uncatalyzed decomposition. This lower activation barrier suggests that modification of Cu‐based catalysts could lead to more optimal reaction conditions.

Catalyst design for alcohol transformation relies on balancing surface acidity and basicity and managing the adsorption strength of alkoxy and hydroxyl groups. Transition metals are effective in dehydrogenation due to their balanced reactivity and stabilization of key intermediates. Cu‐based catalysts are particularly effective, with Cu⁰/Cu⁺ sites facilitating selective alkoxyl activation and Cu^2^⁺ scissoring O─H bonds, while preserving the C─C backbone.^[^ ^237^ ^]^ Copper‐based catalysts supported on ZrO₂ or ZnO enhance the dehydrogenation selectivity of 1,4‐butanediol by stabilizing intermediates and reducing by‐products such as ethers.^[^ ^139^, ^238^ ^]^ Cu/CeO₂─Al₂O₃ also shows excellent performance, achieving 99% selectivity to γ‐butyrolactone at moderate temperatures, attributed largely to the stabilization of key intermediates by basic sites.^[^ ^185^ ^]^ Cu/SiO₂ catalysts achieved a conversion rate of 76% and a selectivity of 94.5% for acetoin from 2,3‐butanediol, with activation energies around 80 kJ mol^−1^.^[^ ^239^ ^]^

Basic sites are well known to support dehydrogenation, with alkali‐earth catalysts like Rb/Al₂O₃ and Sr/Al₂O₃ effectively converting 2‐propanol into propanone.^[^ ^240^, ^241^ ^]^ Similarly, Zn–Mica favors basic sites and selectively dehydrogenates 1‐propanol and 2‐propanol. ZrO₂‐based catalysts illustrate a direct relationship between basic site density and γ‐butyrolactone selectivity in 1,4‐butanediol production.^[^ ^242^ ^]^ Yet in certain catalysts such as alkali‐doped phosphoric acid (M_P/SiO₂, M = Na, K, and Cs), strongly basic sites combine with other active sites to promote dehydrative epoxidation, producing 2,3‐epoxybutane from butanediol.^[^ ^243^ ^]^ Therefore, while basic sites typically enable hydrogen abstraction, their coexistence with acidic or amphoteric sites can redirect the reaction pathway from pure dehydrogenation to dehydration‐based transformations.

In contrast, strongly acidic materials, such as phosphoric acid on silica, aluminosilicates, zeolites, and certain rare‐earth oxides (e.g., Yb₂O₃, Er₂O₃, and CeO₂), promote dehydration, leading to by‐products like tetrahydrofuran, epoxides, or unsaturated alcohols due to abundant Brønsted or Lewis acid sites.^[^ ^243^, ^244^, ^245^, ^246^, ^247^ ^]^ Rare‐earth oxides (e.g., Yb₂O₃, Er₂O₃) exhibit pronounced dehydration activity by coordinating hydroxyl groups and activating β‐hydrogens, which promotes proton elimination or dehydration. Even amphoteric oxides CeO_2_ and Al_2_O_3_ can introduce acidity that encourages dehydration by forming hydrogen bonds with or protonating the hydroxyl group.^[^ ^248^ ^]^ In aqueous‐phase reforming of ethylene glycol, extended residence times on Al₂O₃ demoted dehydrogenation. In contrast, Ru complexes inhibit hydroxyl group adsorption and promote efficient β‐hydride elimination, yielding low dehydrogenation enthalpies around 9.2 kcal/mol.^[^ ^209^ ^]^

### 3.3 Summary and Conclusions for O─H Bonds

O─H bonds are advantageous for hydrogen storage and CO_2_ capture, promoting sustainable energy. Among these, methanol and formic acid stand out for their ease of handling, reasonable hydrogen densities, and the simple dehydrogenation products (CO_2_/CO/H_2_). Formic acid, with a gravimetric density of 4.4 wt.% H and a volumetric density of 53 g H_2_/L,^[^ ^132^, ^133^ ^]^ offers an energy density of 1.8 kWh/L,^[^ ^134^, ^135^ ^]^ surpassing the typical 1.4 kWh/L of 700 bar hydrogen tanks used in fuel cell vehicles such as Toyota Mirai.^[^ ^136^ ^]^ Several heterogeneous and homogeneous catalysts have been developed to allow facile dehydrogenation of formic acid under mild conditions. Methanol, the simplest alcohol, has a hydrogen density of 12.6 wt.% H and 98.8 g H_2_/L, translating to an energy density of 3.26 kWh/L.^[^ ^136^ ^]^ The dehydrogenation of methanol, however, is less straightforward compared with formic acid, which requires higher temperatures and often produces impurities. Higher‐order alcohols face challenges with dehydration, requiring costly catalysts and complex purification.

An intriguing system is the formate‐bicarbonate system, which offers a nearly CO_2_‐ and CO‐free route to hydrogen production, releasing only H_2_ gas.^[^ ^153^, ^154^, ^155^ ^]^ Formates are nontoxic solids that can be easily transported and converted into highly concentrated aqueous solutions. Although their hydrogen energy densities are low (1.2–1.6 wt.% H, 20–28 gH_2_/L), the simplicity and reversibility of the formate–bicarbonate equilibrium make them an attractive hydrogen carrier. Small shifts in reaction conditions, enable efficient cycling near ambient temperatures–ideal for fuel cell applications.^[^ ^153^, ^154^ ^]^

To make O─H systems suitable for practical applications, there needs to be improvement in several aspects. First, more selective catalysts with high stability should be developed. Second, better adsorbents and/or separation of by‐product is needed. Lastly, specialized fuel cells with high tolerance toward CO_2_‐ and CO can be implemented, though their effectiveness may be restricted.

## 4 N─H Bond‐Based Carriers

### 4.1 Choice of Molecules

**Figure 8:**
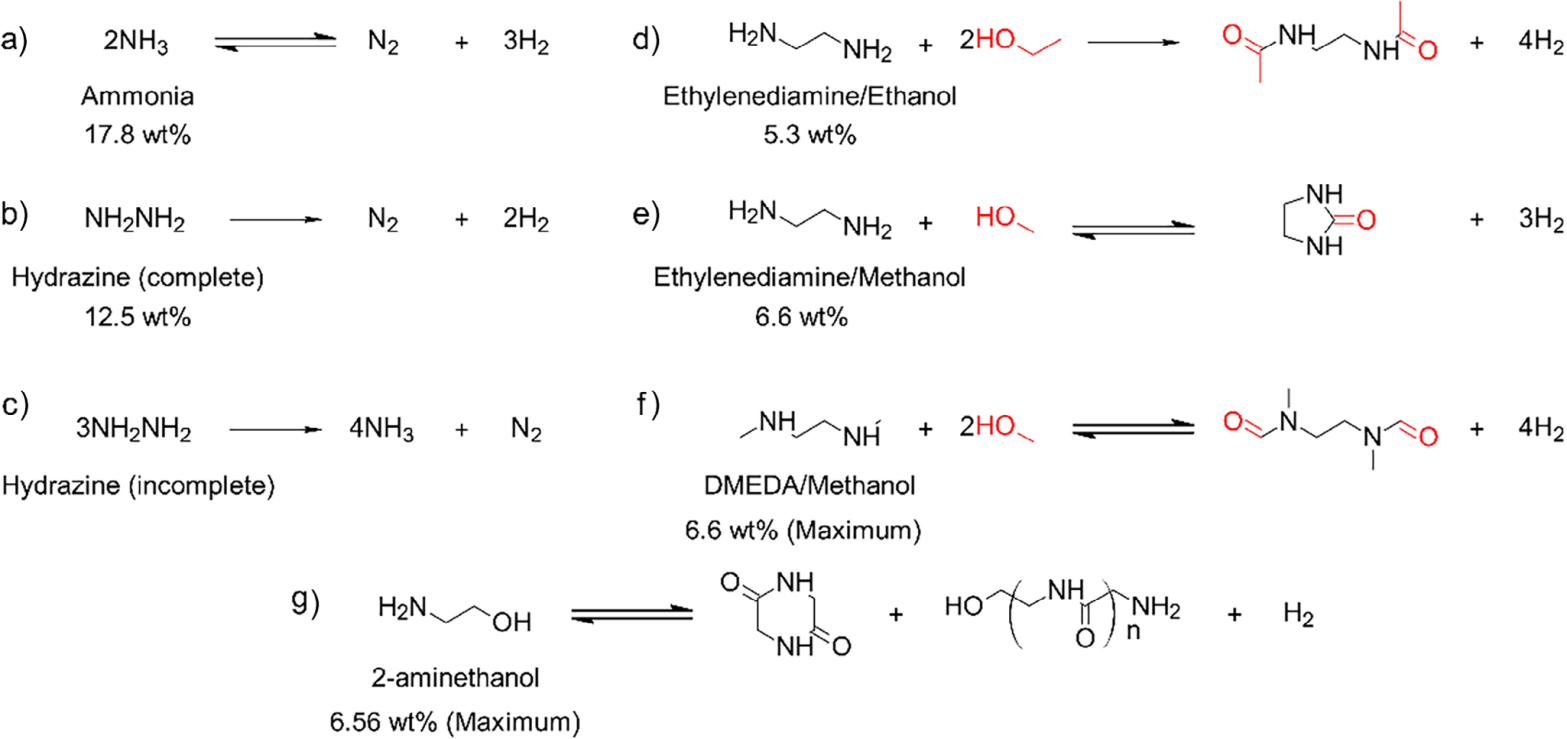
The N─H bond‐based systems for hydrogen storage. In the mixed systems, N─H reacts with O─H (red), driving dehydrogenation.

N─H‐containing compounds are promising hydrogen carriers due to low the BDEs between 370 and 450 kJ mol^−1^. The enthalpy of dehydrogenation for these carriers ranges from 30 to 70 kJ mol^−1^ H_2_, similar to C─H carriers. Ammonia (NH_3_) is a promising hydrogen carrier due to its low cost and well‐established infrastructure,^[^ ^29^, ^249^, ^250^ ^]^ with annual global production between 183 to 225 Mt.^[^ ^251^, ^252^ ^]^ It offers a high volumetric energy density (108 g H_2_/L–NH_3_ at 1 bar and −33.3 °C) and a gravimetric hydrogen density of 17.8 wt.% H, giving it a competitive edge over other carriers. Also, ammonia can be decomposed into only N_2_ and H_2_, avoiding the challenges of carbon‐containing carriers which produce CO and CO_2_ (Figure 8a). Moreover, liquid ammonia has nearly twice the energy density of liquefied hydrogen and requires much milder transport conditions (−33 °C at 1 atm.).

The synthesis of ammonia is well‐known, with the Haber–Bosch process responsible for more than 90% of global production. Despite its exothermic reaction (Δ*H*° = −92.22 kJ mol^−1^), the synthesis of ammonia is energy intensive due to the high activation energy (*E*_a_ = 335 kJ mol^−1^) required to break the N≡N bond (945 kJ mol^−1^).^[^ ^39^ ^]^ Many catalysts, including ruthenium, osmium, and uranium, have been tried to convert N_2_ to NH_3_, but iron‐based catalysts dominate ammonia production with activation energies around 100 kJ mol^−1^.^[^ ^253^, ^254^, ^255^ ^]^ Iron‐based catalysts better facilitate N_2_ chemisorption and nitride (N_3_^−^) formation. Currently, industrial production uses iron‐oxide catalysts and requires temperatures of 400–500 °C and pressures of 150–300 bar.^[^ ^252^, ^256^, ^257^ ^]^ However, the process heavily relies on fossil fuels. Most hydrogen required is derived from natural gas; only 4% is generated via electrolysis.^[^ ^258^, ^259^ ^]^ At best, highly efficient facilities consume approximately 600 kg of natural gas to produce 1000 kg of ammonia.^[^ ^260^ ^]^ Consequently, the annual CO_2_ emissions from ammonia synthesis are estimated to exceed 670 million tonnes, contributing to around 2% of global CO_2_ emissions.

Several promising alternative methods, such as electrochemical and photochemical nitrogen reduction reactions, are being explored to address the energy and emission challenges.^[^ ^261^, ^262^, ^263^, ^264^ ^]^ Another attractive method is to convert nitrates to ammonia electrocatalytically, taking advantage of the cheap and abundant nitrates in wastewater.^[^ ^261^, ^265^ ^]^ Li et al. demonstrated nearly 100% faradaic efficiency in nitrate‐to‐ammonia conversion using ruthenium nanoclusters.^[^ ^265^ ^]^ Readers are directed to the relevant literature for recent developments in this area.^[^ ^266^, ^267^, ^268^, ^269^ ^]^

Hydrogen can be extracted from ammonia using thermal decomposition, laser‐assisted cracking, electrolysis of liquid ammonia, microwave decomposition, plasma treatment, and more.^[^ ^29^, ^270^, ^271^, ^272^, ^273^, ^274^ ^]^ Thermal cracking (or decomposition) relies on the equilibrium of ammonia synthesis, as shown by Equation (23).

(23) $$2{\text{NH}}_{3\left(g\right)}⇋N_{2\left(g\right)}+3H_{2\left(g\right)}\left(\Delta H=+92.44\;\text{kJ}\;{\text{mol}}^{-1}\right).$$

**Figure 9:**
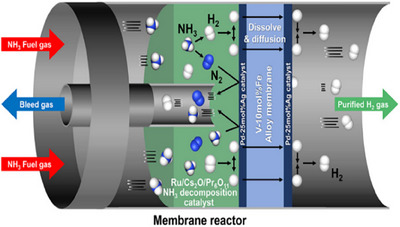
Graphical representation of an ammonia membrane reactor to produce high‐purity hydrogen.^[^ ^275^ ^]^

The decomposition reaction reaches an equilibrium, requiring specific pressure and temperature for high hydrogen conversion (>99%). High pressure requires a high temperature to counter the reverse reaction, while lower pressure allows for lower temperatures. At atmospheric pressure, temperatures over 400 °C are needed, whereas at 40 bar 700 °C is necessary to achieve quantitative conversion. Incomplete conversion results in ammonia impurities in the hydrogen stream, necessitating postpurification methods such as PSA or H_2_‐permeable membranes. Innovations in catalysts and reactor engineering are vital to use ammonia as a hydrogen carrier. Catalytic membrane reactors (CMRs) efficiently split ammonia into hydrogen and nitrogen at various scales.^[^ ^275^, ^276^ ^]^ They consist of a catalyst and a selective permeable membrane that continuously filters hydrogen, shifting the equilibrium toward nitrogen and hydrogen and enhancing ammonia conversion by preventing reverse reactions (Figure 9). This integration makes CMRs ideal for producing high‐purity hydrogen compactly.

**Figure 10:**
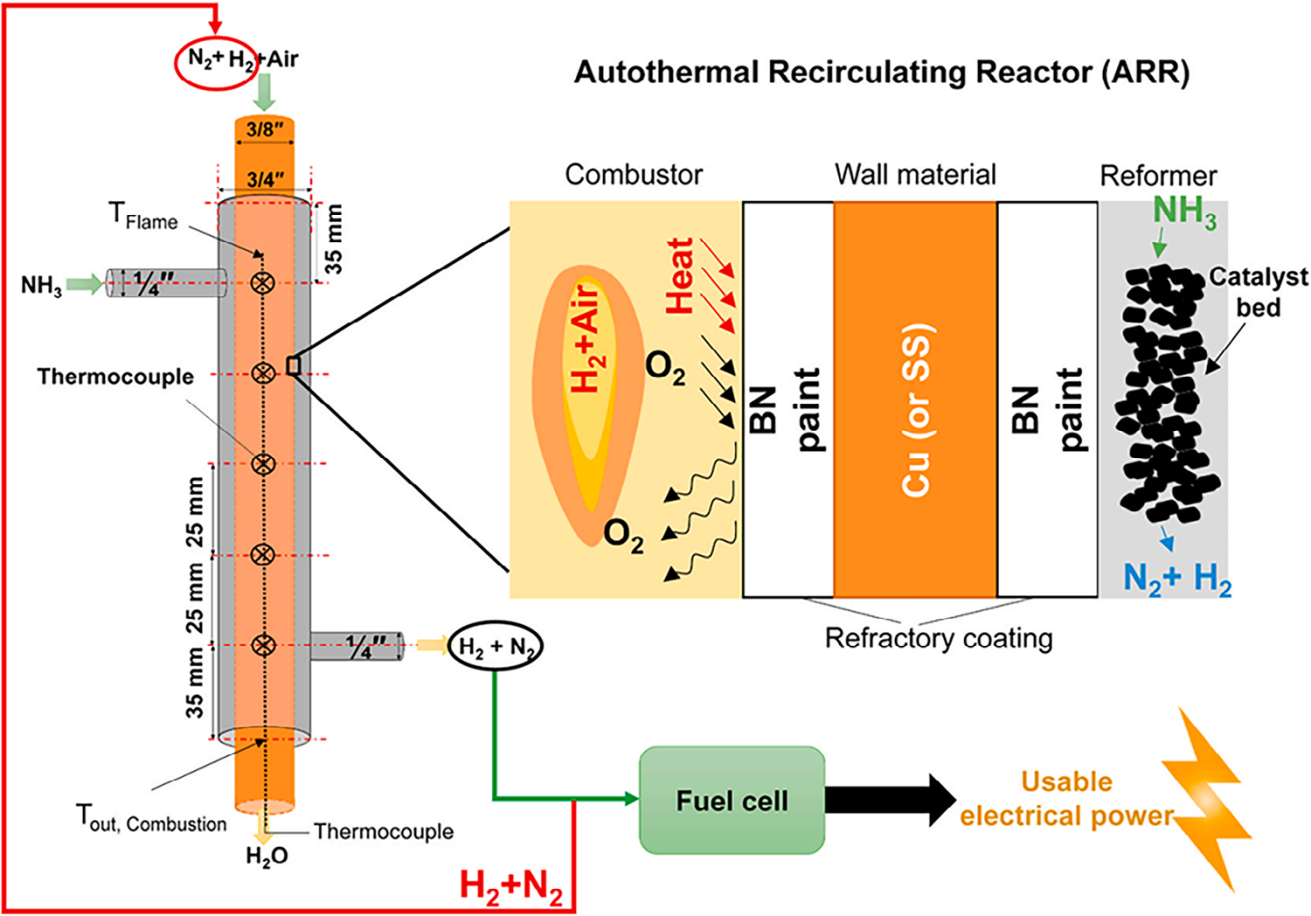
An autothermal recirculating reactor for power generation from ammonia decomposition.^[^ ^285^ ^]^

Microreactors also offer a compact solution, especially for mobile applications, by combining ammonia decomposition with exothermic reactions and using catalysts such as ruthenium or platinum. Early designs utilized butane combustion for heat, ^[^ ^277^ ^]^ but recent innovations emphasize ammonia‐based oxidation and decomposition to eliminate CO_2_ emissions.^[^ ^278^, ^279^ ^]^ Autothermal microchannel reactors have been developed using stainless steel plates coated with Ru and Pt catalysts on alumina supports. These reactors enhance conversion and reduce energy demands through heat recycling.^[^ ^273^, ^278^, ^279^, ^280^, ^281^, ^282^, ^283^, ^284^ ^]^ Jo et al. created an autothermal recirculating reactor (ARR) for ammonia decomposition, achieving 99.6% NH_3_ conversion and 70.95% reforming efficiency (Figure 10).^[^ ^285^ ^]^ Their double‐tube module allows for partial hydrogen recirculation for combustion, generating 84 W of fuel cell power without external heat sources. They identified stainless steel as suitable and found boron nitride‐coated copper composite with better heat transfer.

Electrocatalytic ammonia decomposition is a promising method for sustainable hydrogen production, especially given its advantages over traditional water electrolysis. The ammonia electro‐oxidation reactor (AOR) converts ammonia into hydrogen and nitrogen or nitrates with low theoretical energy input. This can result in great energy efficiency (33 MJ/kg H_2_ for AOR vs. 180 MJ/kg H_2_ for water splitting).^[^ ^286^, ^287^, ^288^ ^]^ Platinum group metals (Rh, Pt, and Ir) are effective AOR catalysts,^[^ ^266^, ^287^, ^288^, ^289^, ^290^, ^291^, ^292^ ^]^ achieving over 90% faradic efficiency when electroplated onto carbon fibre substrates.^[^ ^293^ ^]^ Jacob et al. recently reported a Ru complex ([Ru(bipyridinedicarboxylate)(4‐methylpyridine)_2_]) that achieved over 80% Faradic efficiency with a high TOF of 3757 s^−1^ (13525198 h^−1^).^[^ ^294^ ^]^ However, its high cost limits large‐scale use. Researchers are exploring more affordable options such as Ni‐based catalysts, including Ni oxides, Ni hydroxides, and Ni oxyhydroxides, which also show promising performance for AOR.^[^ ^268^, ^287^, ^295^ ^]^

Anhydrous hydrazine (H_2_NNH_2_, AH) is a valued N─H carrier in aerospace due to its hypergolic properties. It powered missions like Apollo 9 and the Mars Rover.^[^ ^296^, ^297^, ^298^ ^]^ It has a high hydrogen content (12.5 wt.% H and 129 g H_2_/L). Complete decomposition yields N_2_ and H_2_ (Figure 8b), while incomplete decomposition forms ammonia (Figure 8c).^[^ ^299^, ^300^, ^301^, ^302^, ^303^ ^]^ Although direct fuel cells using anhydrous hydrazine face safety issues, including toxicity and explosion risks, hydrazine hydrates (e.g., H₂NNH₂·H₂O) offer a safer alternative with good hydrogen capacity (8.0 wt.% H) and compatibility with existing refuelling infrastructure.^[^ ^304^, ^305^, ^306^, ^307^, ^308^, ^309^ ^]^

Amines are also considered for hydrogen storage but struggle with efficient hydrogen release due to their structure. Like LOHCs, they need a catalyst for H₂ evolution. Combining amines with alcohols enhances this process by promoting hydrogen abstraction and forming reducible amides and related products (Figure 8d–f). These systems could scale up effectively with cost‐efficient hydrogen reactors, utilizing low‐cost amines and alcohols. Moreover, they support carbon neutrality, as carbon remains sequestered in stable *N*‐formamides or urea, preventing gaseous carbon release.

The Hu group's research highlights the effectiveness of dehydrogenating ethylenediamine (EDA) with ethanol, achieving a hydrogen capacity of 5.3 wt.% H at 135 °C using 0.2 mol% catalyst (Figure 8d).^[^ ^310^ ^]^ This process produced 95% hydrogen and 93% *N*,*N*'‐diacetylethylene diamine (DAE), with 99% recovery of EDA and 89% EtOH under 70 bar H₂. Similar studies using methanol instead of ethanol demonstrated challenges, as intermediates such as formamide, diformamide, and trace amounts of CO were detected.^[^ ^24^ ^]^ The Kothandaraman group's exploration of amine alcohol systems led to two recyclable systems. Using homogenous ruthenium catalysts, ethylenediamine and methanol were converted into cyclic urea (Figure 8e).^[^ ^9^ ^]^ Combining *N*,*N*’‐dimethylethylenediamine (DMEDA) and methanol produced *N*,*N*’‐(ethane‐1,2–diyl)bis(*N*‐methylformamide) (EMFA) and hydrogen (Figure 8f), achieving up to 86% of the theoretical gravimetric hydrogen capacity. Regeneration under 60 bar H₂ with a ruthenium pincer complex recovered 95% of DMEDA, though toxicity and flammability remain concerns. Additionally, Mn‐based catalysts showed high selectivity (97%).^[^ ^311^ ^]^

Alkanolamines such as 2‐aminethanol can dehydrogenate to yield either piperazine‐2,5‐dione (glycine anhydride, GA) or linear oligopeptides, depending on conditions (Figure 8g).^[^ ^312^ ^]^ Forming GA leads to a high hydrogen capacity of 6.56 wt.% H, exceeding the 5.46 wt.% H in the case of oligopeptides. Using a ruthenium pincer catalyst and KOtBu, a system achieved an 85% conversion of 2‐aminethanol, yielding 60% GA and 77% hydrogen. Hydrogenation under 50 bar H_2_ at 110 °C regenerated 81% of 2‐aminoethanol after multiple cycles without extra catalyst input. For more details, it is recommended to consult the relevant literature.^[^ ^313^, ^314^, ^315^ ^]^ Table 6 summarizes the physical and thermodynamic properties of the N─H‐based hydrogen carriers discussed in this section.

**Table 6:** Physical and thermodynamic properties of the common N─H‐based carriers.^[^ ^29^, ^39^, ^249^, ^250^, ^299^, ^300^, ^301^, ^302^, ^303^, ^310^ ^]^

	Melting Points (°C)		Boiling Points (°C)					
Chemical	H_2_‐Rich	H_2_‐Lean	H_2_‐Rich	H_2_‐Lean	Bond Length (Å)	BDE (kJ mol^−1^)	Enthalpy (kJ mol^−1^, kJ mol^−1^ H_2_)	Hydrogen Capacity (wt.% H, gH_2_ L^−1^)
Ammonia	−77.73	–	−33.34	–	1.012	450	92.4, 30.81	12.6, 98.8
Hydrazine	114	–	2	–	1.021	450	–	12.5, 129
Ethylene diamine	8	–	116	–	1.010	–	–	4.34–6.52, –

### 4.2 Catalysts: Established and Emerging Choices

**Figure 11:**
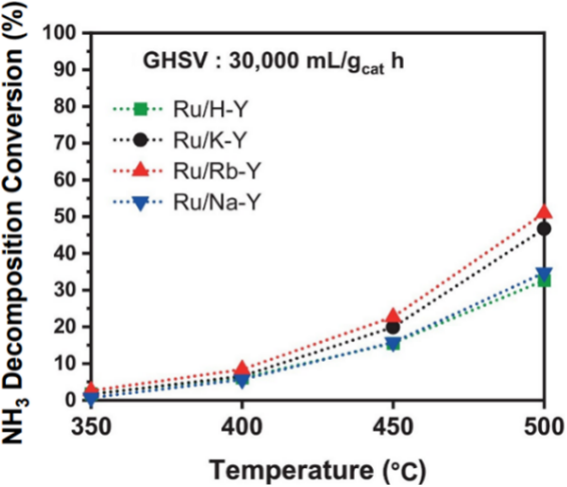
Catalytic activity for ammonia decomposition at 350–500 °C, over Ru/H‐Y, Ru/K‐Y, Ru/Rb‐Y, and Ru/Na‐Y at 30 000 mL/g_CAT_‐GHSV.^[^ ^329^ ^]^

Ammonia's promising hydrogen storage properties have spurred research into innovative catalysts. Various metals and alloys have been explored, including FeOsx,^[^ ^316^, ^317^ ^]^ Fe‐Mn,^[^ ^318^ ^]^ Ni, Co,^[^ ^319^, ^320^ ^]^ Ir, Rh, Pd, Pt, Mo,^[^ ^321^ ^]^ Cu, Ru,^[^ ^322^ ^]^ and Cr.^[^ ^29^, ^273^, ^323^, ^324^ ^]^ Some found that catalytic activity follows the order of Ru > Ni > Rh > Co > Ir > Fe > Pt > Cr > Pd > Cu >> Te, Se, and Pb,^[^ ^325^ ^]^ while others found Ir is more effective than Rh.^[^ ^273^, ^326^, ^327^, ^328^ ^]^ The most reported catalysts are Ni and Ru, with Ni being preferred due to its low cost, wide range of activity and lifespan. Cha et al. synthesized nanometer‐sized Ru particles on various zeolite Y supports (Ru/H‐Y, Ru/Na‐Y, Ru/K‐Y, and Ru/Rb‐Y) through ion‐exchange and vacuum calcination (Figure 11).^[^ ^329^ ^]^

Ru/Rb‐Y exhibited the highest catalytic activity due to increased basicity at the Ru sites, which improves nitrogen desorption and is crucial for efficient ammonia decomposition. This contradicts the acidity trend of the support, indicating that the high basicity of Rb‐Y is key.

Currently, ammonia decomposition in commercial settings commonly uses Ni supported by alumina (Ni/Al_2_O_3_), providing effective conversion at temperatures of 400–600 °C and pressures of 10–40 atm.^[^ ^323^ ^]^ Table 7 summarizes some of the recent and best‐performing catalysts for N─H bond activation.

**Table 7:** Summary of the recent and best‐performing catalysts for N─H bond activation.

Reactant	Catalyst	Temperature (°C)	Loading	TOF (h^−1^)	Results	*E*_a_ (kJ mol^−1^)	Ref.
Ammonia	10 wt.% Ni/SiO_2_	600	–	42 912	–	–	[330]
	10 wt.% Ru/SiO_2_	600	–	160 992	Conversion = 97%	–	[330]
	Mo_2_C	570	–	101 880		173.4	
	1.8 wt.% Ru/La_0.33_Ce_0.67_	400	–	9720	Performed > 100 h	80	[331]
	Co_0.89_Fe_2.11_O_4_@mSiO_2_	450	50 mg_CAT_, 19 mL_NH3_ min^−1^	583	–	126.4	[332]
	3.0 wt.% Ru/Ba‐ZrO_2_	500	–	–	Conversion = 37.8% 12.7 mmol_H2_ g^−1^ min^−1^	64.2	[333]
	2.5 Ni_0.5_Ru/CeO_2_	400	0.1 g_CAT_, 25 mL_NH3_ min^−1^	7, 200	50% conversion at 400 °C, 99.2% conversion at 500 °C	107	[334]
	5 wt.% LiNH_2_‐Ru/MgO	547	30 mg_CAT_, 30 mL_NH3_ min^−1^ (5 vol%)	–	Conversion = 100%, 68.3 mmol_H2_ g^−1^ min^−1^	53.2	[335]
	5.5 wt.% MRM‐Ru‐R	700	12 h, 25 mg_CAT_, and 4000 mL_NH3_ min^−1^	–	99.6% conversion, 240 mmol_H2_ g^−1^ min^−1^	91.7	[336]
	Ru/Rb‐Y	500	80 mg_CAT_, 500 mL_NH3_ g_CAT_^−1^ min^−1^	5, 279	–	78.5	[329]
Ethylenediamine + Alcohol	RuHCl(^tBu^PNNH)(CO)	150	24 h, 0.01 mmol_CAT_, 1 mmol_EDA_, and 3 mmol_MeOH_	–	99% conversion, 76% selectivity to ethylene urea	–	[24]
	RuH(Cl)(PNN)(CO)]	135	0.01 mmol_CAT_, 5 mmol_EDA_, and 12 mmol_EtOH_	–	100% EtOH conversion, 93% H_2_ yield	–	[310]
Hydrous Hydrazine	Rh–Ni/Ce(OH)CO_3_	30		150	100% H_2_ selectivity	38.8	[337]
	PtNi/PDA‐rGO	30	3 min, 0.1 mmol_CAT_	903	100% H_2_ selectivity	33.4	[338]
	PtNi‐CNDs	50	7 min, 0.2 mmol_CAT_	594	100% H_2_ selectivity	43.9	[339]

CMRs offer an efficient solution for hydrogen production by combining catalytic decomposition and purification. Omata et al. designed a CMR that uses a Ru/Cs_2_O/Pr_6_O_11_ decomposition catalyst and a 10 mol% vanadium iron alloy membrane (Figure 9), achieving over 3000 h of continuous operation and 80% ammonia conversion at fuel cell‐grade purity (NH₃ < 0.1 ppm; N₂ < 100 ppm, and ISO 14 687–2).^[^ ^275^ ^]^ Although palladium alloys are typically preferred for hydrogen permeability, vanadium was chosen for its cost‐effectiveness.^[^ ^340^ ^]^ However, vanadium's toxicity and carcinogenicity have led to its prohibition in many countries, necessitation alternatives.^[^ ^341^, ^342^ ^]^

Hydrogen‐permeable membranes are essential for CMR performance. Membranes suitable for industrial use must have high hydrogen permeability, chemical and thermal stability, resistance to poisoning, and compatibility with catalysts. Cost efficiency and scalability are also essential. Nickel‐based alloys offer lower costs with moderate permeability.^[^ ^343^, ^344^ ^]^ Ceramic membranes such as silicates and zirconia are stable under extreme conditions but need improved hydrogen permeability. Composite membranes combining metal and ceramic properties provide a good balance of cost and performance.^[^ ^345^, ^346^, ^347^, ^348^, ^349^ ^]^ Advances in nonprecious metal catalysts and scalable membrane fabrication could further reduce costs.

There are also novel approaches aimed at reducing the cost of hydrogen production from ammonia using low‐cost catalysts and supports. Researchers explored cost‐effective methods to produce hydrogen from ammonia using red mud, an iron oxide‐rich industrial by‐product.^[^ ^350^, ^351^ ^]^ Red mud shows strong catalytic performance in ammonia decomposition, maintaining over 72 h of efficiency at 700 °C, similar to ruthenium‐based systems. Modified red mud catalysts with ruthenium can operate for 7 days at this temperature. With around 120 million tonnes produced annually from aluminium manufacturing, red mud offers a viable option for large‐scale hydrogen production.^[^ ^352^, ^353^, ^354^ ^]^ This approach supports circular economy by repurposing hazardous waste and minimizing landfill use. Other novel nonmetal‐based catalysts, such as a geometrically constrained phosphine organocatalyst, have shown activity toward N─H bond cleavage.^[^ ^355^ ^]^

Catalytic ammonia decomposition initially involves the adsorption of NH₃ onto catalyst surface, followed by sequential N─H bond cleavage. The adsorbed N and H species then undergo associative desorption reactions, producing H_2_ and N_2_. Noble metals typically have N─H cleavage as the rate‐limiting step, while for non‐noble metals nitrogen recombination is more critical. Optimizing M─N bond strength is essential for efficient N─H cleavage and N₂ release.^[^ ^322^, ^356^ ^]^

Support and microstructure can shift the reaction dynamics. Taking Ru catalysts as an example, if associative N_2_ desorption is the rate‐determining step, weakening the Ru─N bond strength is essential. This can be achieved by adding promotors that increase metal electron density, thereby populating the π* bonding orbital of the Ru─N bond. Additionally, modifying the surface properties of the support (acidity/basicity) also influences this process. Local structure is also crucial to promote NH_3_ dehydrogenation. Ru B_5_ sites are known to accelerate this process. A recent study utilized hexagonal boron nitride (h‐BN) sheets as a template support and leveraged the h‐BN(001) surface to facilitate the formation of Ru B_5_ sites, which exhibited enhanced activity for low‐temperature ammonia dehydrogenation.^[^ ^357^ ^]^

In general, supports are crucial for dispersing active sites, enhancing stability, and modifying surface properties. Alkaline additives and basic supports that boost electron density improve ammonia decomposition by enhancing nitrogen recombination and desorption rates.^[^ ^322^, ^358^ ^]^ Additionally, waste‐derived materials such as red mud and fly ash offer cost‐effective and sustainable alternatives.^[^ ^358^ ^]^

Ru catalysts are benchmarks due to their exceptional activity and durability, but their high cost has prompted the search for alternatives, such as bimetallic iron‐cobalt, which show promise in laboratory studies.^[^ ^359^ ^]^ Techniques such as steady‐state isotopic transient kinetic analysis help investigate surface interactions, offering insights into reaction mechanisms and guiding future catalyst design.^[^ ^322^ ^]^ For a detailed exploration of ammonia decomposition mechanisms and catalyst advancements, readers are referred to these publications.^[^ ^356^, ^359^ ^]^

The century‐old ammonia industry, already producing around 183 to 225 Mt annually,^[^ ^251^, ^360^ ^]^ presents an attractive case for adopting ammonia as a chemical hydrogen carrier. Therefore, the regeneration of N─H bond‐based hydrogen carriers at an efficient large‐scale process is effectively limited to ammonia. Techno‐economic analysis has shown that the cost of hydrogen production from ammonia varies significantly, ranging from USD 3.6 to 7.4 per kg of hydrogen, while the price of ammonia ranges from USD 130 to USD 910 per ton.^[^ ^27^, ^251^, ^258^, ^361^, ^362^, ^363^, ^364^, ^365^ ^]^ Lin et al. found that ammonia feed accounts for about 74% of the hydrogen production cost.^[^ ^366^ ^]^ Microreactors, while offering a compact and efficient solution for ammonia decomposition, have been estimated to produce hydrogen at much higher costs, ranging from USD 167 to USD 333 per kg of hydrogen.^[^ ^36^ ^]^

To lower costs and address environmental impact, several alternative methods for ammonia synthesis, such as electrochemical and photochemical nitrogen reduction reactions (NRRs), are being explored. The major challenge of NRRs is breaking the strong N≡N bond, which has a large BDE of 945 kJmol^−1^.^[^ ^39^ ^]^ Lithium‐mediated NRR has shown promise for efficiently cleaving the N≡N bond.^[^ ^262^, ^263^, ^264^ ^]^ In the Li‐NRR process, lithium metal cleaves the N≡N triple bond, forming a nitrogenous lithium compound that is subsequently converted into ammonia. Although early results have shown promise, faradic efficiencies remain below 20%.^[^ ^367^ ^]^

Ammonia is less flammable than gasoline and natural gas.^[^ ^249^ ^]^ However, its use as an energy carrier raises challenges due to its toxicity.^[^ ^266^, ^368^ ^]^ Ammonia has been normally transported in large quantities via pipelines and tankers. To minimize environmental impact and health impact, ammonia handling requires experience and rigorous training in a highly organized and centralized way. Conventional ammonia thermal cracking is energy intensive and requires strong safety protocol. In comparison, electrolysis of ammonia–water solution produces hydrogen at room temperature and the process can be carried out in a decentralized way due to the low‐risk profile of this technology.

### 4.3 Summary and Conclusion for N─H Bonds

Ammonia is the most studied N─H containing compound for hydrogen storage. It has higher hydrogen capacity than liquid hydrogen and most hydrogen carriers per unit mass and per unit volume. It becomes liquid at temperatures below −33 °C at atmospheric pressure or liquefies at 20 °C at 7.5 bar. Its wide availability and very established infrastructure, makes it easy to deploy ammonia globally as a reliable hydrogen carrier. However, its (de)hydrogenation processes require significant energy for both thermal decomposition and gas separation, creating economic barriers.

For ammonia to play a role as an energy carrier in sustainable future, its synthesis needs to be improved. Current ammonia synthesis consumes around 1%–2% of the world's total energy production, primarily due to the energy‐intensive Haber–Bosch process, which relies heavily on fossil fuels to generate the necessary hydrogen for the reaction. As a result, its synthesis releases 2% global CO_2_ emissions. Renewable hydrogen has been tested to produce green ammonia, which can reduce carbon footprint. In addition, we can advance electrochemical and photochemical nitrogen reduction processes and develop cost‐effective catalysts to find more energy efficient method to produce ammonia.

## 5 B─H Bond‐Based Hydrogen Carriers

### 5.1 Choice of Molecules

B─H bond‐based hydrogen carriers are known for their high hydrogen capacity and unique chemical behavior. The small electronegativity difference between boron (2.04) and hydrogen (2.20) results in hydridic hydrogen, distinct from protic hydrogens in O─H, N─H, and C─H bonds. Boron's empty p orbital allows for various B_m_H_n_ structures, with metal borohydrides and amine boranes being the most studied. Their decomposition pathways are unique compared to other X─H bond‐based hydrogen carriers. Measuring the BDE of B─H bonds is challenging, with values varying from 190 to 470 kJ mol^−1^ and bond lengths from 1.07 to 1.27 Å.^[^ ^39^, ^369^, ^370^, ^371^, ^372^ ^]^ Rablen and Hartwig found that the first B─H cleavage has a BDE of 432.6 to 459.8 kJ mol^−1^ (103.4 to 109.9 kcal/mol), comparable to or stronger than C─H bonds.^[^ ^369^, ^370^, ^371^ ^]^ Subsequent cleavages have weaker BDEs, around 334.7 kJ mol^−1^ (80 kcal/mol), and the overall BDEs for organoboranes tend to be lower than hydrocarbons. B─H containing compounds can form coordinate covalent (dative) bonds with Lewis bases such as NH_3_ and amines.^[^ ^373^, ^374^, ^375^ ^]^ The strong interaction between hydridic and protic hydrogens, indicated by short bond distances of 2.02–2.22 Å,^[^ ^376^, ^377^, ^378^, ^379^ ^]^ facilitates dihydrogen release, contrasting with longer bond length (2.35 to 2.48 Å) found in Ar–H**···**H–Ar (Ar = aromatic) interactions.

**Figure 12:**
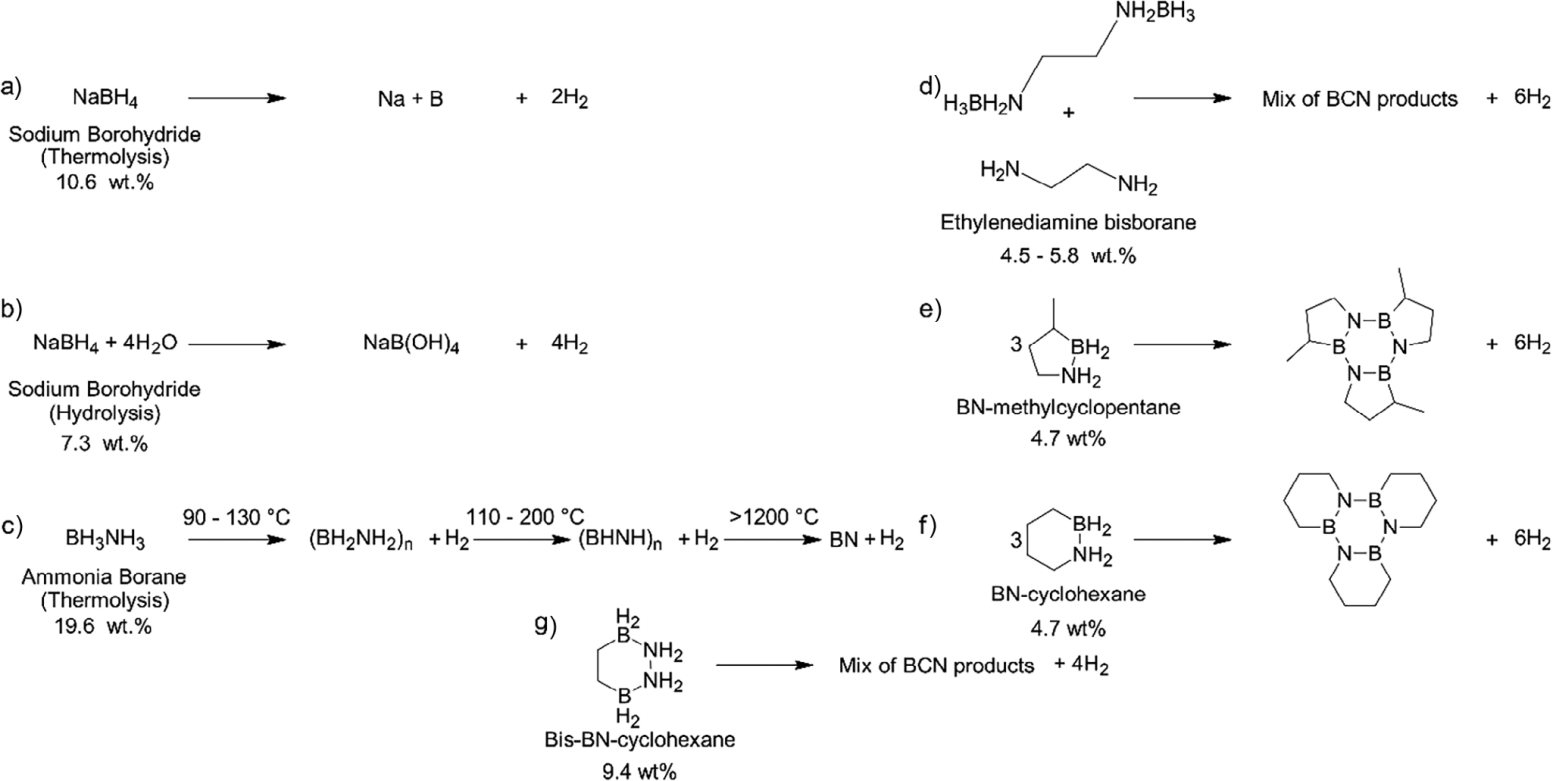
The B─H bond‐based systems for hydrogen storage.

B─H containing compounds such as ammonia borane (NH_3_BH_3_) and borohydrides have been studied extensively for hydrogen storage (Figure 12).^[^ ^34^ ^]^ Borohydrides (BH_4_^−^) feature high hydrogen densities, and their solid‐state nature ensures lower volatility and therefore enhanced safety compared with some liquid carriers. These materials are also relatively stable under ambient conditions, which makes them suitable for transport and storage without extensive containment systems.^[^ ^380^, ^381^ ^]^ The strength of interaction between the metal cation and the borohydride anion determines the stability and decomposition temperatures of borohydrides. Metals with higher charge densities, higher positive charges, and smaller ionic sizes polarize the borohydride ion more effectively. This increased polarization weakens the B─H bonds, leading to lower decomposition temperatures and facilitating efficient hydrogen release.^[^ ^382^, ^383^ ^]^ This explains alkaline earth metal borohydrides such as magnesium (Mg^2^⁺, *χ*_p_ = 1.31) and calcium (Ca^2^⁺, *χ*_p_ = 1.00) decompose more readily than those of alkali metals such as lithium (Li⁺, *χ*_p_ = 0.98) and sodium (Na⁺, *χ*_p_ = 0.93).^[^ ^39^ ^]^

Borohydrides release hydrogen via thermolysis or hydrolysis (Figure 12a,b).^[^ ^384^, ^385^ ^]^ For example, LiBH_4_ has a gravimetric hydrogen density of 18.3 wt.% H and releases hydrogen at 280 °C, but rehydrogenation requires severe conditions (600 °C, 850 bar H_2_).^[^ ^33^, ^386^, ^387^, ^388^ ^]^ Its practical desorption is limited to 13.8 wt.% H due to LiH formation, which needs 700 °C to release hydrogen. The B─H bond lengths of 1.21–1.22 Å indicate moderate bond strength, with dehydrogenation enthalpies ranging from 56 to 75 kJ mol^−1^ H₂.^[^ ^39^, ^389^, ^390^, ^391^ ^]^ NaBH_4_, with stronger B─H bonds of 1.16 Å,^[^ ^392^ ^]^ releases hydrogen at 565 °C with a capacity of 10.6 wt.% H and higher enthalpies (90–108 kJ mol^−1^H_2_).^[^ ^393^, ^394^ ^]^ Mg(BH_4_)_2_ has bond lengths between 1.17 and 1.28 Å and a hydrogen density (14.9 wt.% H, 145–147 gH_2_/L) but offers a lower dehydrogenation temperature of around 320 °C.^[^ ^35^ ^]^ Hydrolysis provides an alternative hydrogen release pathway, particularly for NaBH_4_ (Figure 12b), with a capacity of 7.3 wt.% H and a spontaneous reaction (Δ*H* = −240 kJ mol^−1^) under ambient conditions.^[^ ^395^ ^]^

**Figure 13:**
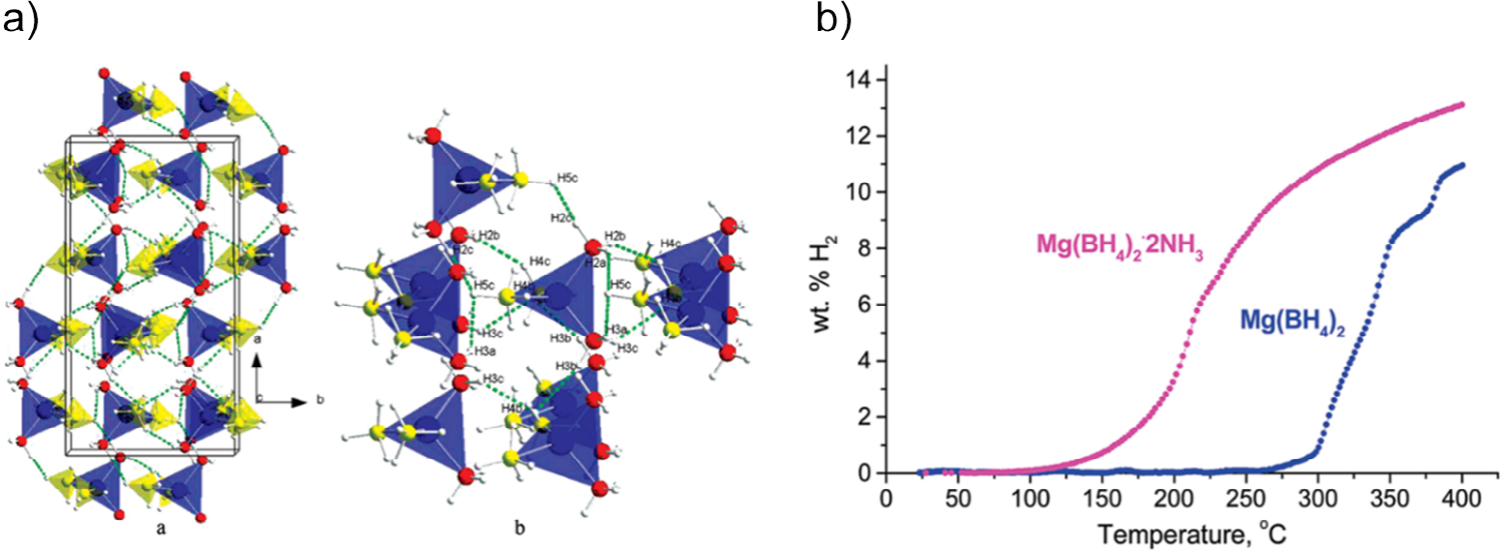
A) Molecular packing a) and network of N─H···H─B dihydrogen bonds b) in the crystal structure of Mg(BH_4_)_2_·2NH_3_. Mg atoms are depicted as blue, N atoms are red, B atoms are yellow, and H atoms are white. H···H interactions of less than 2.33 Å are shown as dashed green lines. (B) Gas evolution from Mg(BH_4_)_2_·2NH_3_ (magenta line) and Mg(BH_4_)_2_ (blue line).^[^ ^8^ ^]^

Researchers have explored composites of metal borohydrides with metal hydrides, metal oxides, or amides to adjust thermodynamic stability and lower desorption temperatures (Table 8).^[^ ^396^, ^397^, ^398^, ^399^, ^400^, ^401^, ^402^, ^403^ ^]^ Reactive hydride composites (RHCs) feature thermodynamics different from borohydrides through chemical reactions during thermolysis.^[^ ^404^ ^]^ A notable example is the 6LiBH₄–CaH₂–MgH₂ system, which offers ∼8.0 wt.% H reversible hydrogen capacity at 400 °C under 100 bar H_2_. Hydrogen release started at 290 °C, 160 °C lower than 6LiBH₄‐CaH₂ alone, which was driven by a 12% reduction in activation energy from MgH₂.^[^ ^7^ ^]^ Nanoscale MgH_2_ has improved storage performance due to better interfacial interactions. Nanoconfinement can improve reaction rates and facilitate hydrogen release at lower temperatures.^[^ ^405^, ^406^, ^407^, ^408^, ^409^ ^]^ Borohydride ammoniates such as Mg(BH₄)₂·2NH₃ utilize N─H^σ+^···^σ⁻^H─B dihydrogen bonds for hydrogen storage, delivering 16 wt.% H, with decomposition beginning endothermically at 150 °C (Figure 13).^[^ ^8^ ^]^ Similarly, Ca(BH₄)₂·2NH₃ releases 11.3 wt.% H at 250 °C driven by dihydrogen bonds with distances ranging from 2.009 to 2.352 Å.^[^ ^410^ ^]^

**Table 8:** Hydrogen storage properties of different borohydrides, adapted from Ref. [411] and [412].

Material	Hydrogen Capacity (wt.% H)	Melting Point (°C)	Decomposition Temperature (°C)	Enthalpy of Formation (kJ mol^−1^ H_2_ or *BH_4_)	Desorption Enthalpy (kJ mol^−1^ H_2_)	Entropy (J mol^−1^ K)
LiBH_4_	18.3	284	380	∼ −180	56–75	76–238
NaBH_4_	10.6	505	565	∼ −190	90–108	101–133
KBH_4_	7.4	500	584	∼ −230	–	106–162
Mg(BH_4_)_2_	14.9	320	320	−99*	39–57	9–128
Ca(BH_4_)_2_	11.5	320	347	−151*	75.5–87	158
Zn(BH_4_)_2_	8.4	85	85	−18*	–	–
Sc(BH_4_)_3_	13.5	–	260	−106*	–	–
Al(BH_4_)_3_	16.9	−64 (volatile)	44.5	−301.8	6	289
Zr(BH_4_)_4_	10.7	–	250	−87*	–	–

Ammonia borane (AB) is notable for its high hydrogen content (19.6 wt.% H) and density (151 g H_2_/L), making it an appealing hydrogen carrier.^[^ ^2^, ^377^, ^413^ ^]^ Although AB and ethane (CH₃─CH₃) are isoelectronic, they differ in physiochemical behavior. AB has a dative, polar B─N bond, while ethane features a covalent, nonpolar C─C bond. The B─H bonds in AB measure 1.08–1.13 Å, with BDEs ranging from 71.5 ± 3 kJ mol^−1^ for cationic states to 419 ± 10 kJ mol^−1^ for the neutral molecule, compared to ethane's stable C─H bond at 1.09 Å and BDE of 410 kJ mol^−1^.^[^ ^39^, ^377^ ^]^ Despite its favorable hydrogen storage, AB releases volatile impurities such as ammonia and borazine upon decomposition, which reduces usable hydrogen content and complicates handling. Additionally, spent fuel produces stable BN_x_ (thermolytic product) or BO_x_‐based oxides (hydrolytic product), which pose a significant barrier to regeneration, requiring extensive energy input to revert to AB (Figure 12c). The efficiency in converting BN_x_ to AB is crucial. The typical digestion‐reduction‐ammoniation route, though effective, is costly and complex due to its dependence on strong reducing agents, such as LiAlH₄, to regenerate the BH₃ unit. Alternatively, Sutton et al. developed a more practical one‐step regeneration process using hydrazine in liquid ammonia at 40 °C for 24 h, achieving near‐complete reformation. Although this method represents a step toward sustainable AB cycling, challenges like volatile by‐products and high material costs remain.^[^ ^414^, ^415^, ^416^, ^417^, ^418^, ^419^ ^]^ Several strategies have been proposed to address the challenges of AB as a hydrogen carrier. Common approaches include using aprotic solvents^[^ ^420^ ^]^ or ionic liquids^[^ ^421^ ^]^ to suppress by‐products, alongside solid‐state doping,^[^ ^422^ ^]^ nanoconfinement,^[^ ^423^ ^]^ or chemical modification to enhance hydrogen release kinetics and purity.^[^ ^424^ ^]^

Alkylamine boranes, derivatives of AB, have improved dehydrogenation kinetics and reduced volatile impurities. These derivatives disrupt dihydrogen bonding, lowering exothermicity and activation energy while stabilizing the B─N bond. Ethylenediamine bisborane (EDAB) delivered high‐purity hydrogen release (up to 9.4 wt.% H) without an induction period.^[^ ^425^, ^426^, ^427^, ^428^ ^]^ A liquid‐phase system using EDAB with ethylenediamine (EDA) and a Pt/C catalyst can produce up to 8.2 wt.% H (Figure 12d), with improved regeneration through simple reactions with NaBH₄ and water at ambient conditions.^[^ ^429^ ^]^ Zhang et al. studied polyamine‐boranes like DETAB (C₄H₁₃N₃ • 3BH₃), TETAB (C₆H₁₈N₄ • 4BH₃), and TEPAB (C₈H₂₃N₅ • 5BH₃), finding their thermal decomposition occurs in two steps: between 90–150 °C and 170–230 °C, similar to EDAB's dehydrogenation pathway.^[^ ^430^ ^]^ This process eliminates by‐products such as borazine and ammonia, resulting in cleaner hydrogen release. TEPAB shows higher reactivity with an activation energy of 61.19 kJ mol^−1^ due to destabilization from its extended carbon backbone. These findings highlight that fine‐tuning alkylamine boranes could enhance hydrogen storage technologies, making optimization of their capacity and kinetics central to unlocking their practical application.

Additionally, octahydrotriborates, such as NaB₃H₈ and NH₄B₃H₈ offer high‐purity hydrogen release during hydrolysis, although pyrolysis can introduce minor borane impurities like B₂H₆ and B₅H₉.^[^ ^431^, ^432^, ^433^ ^]^ Chen et al. identified guanidinium octahydrotriborate as an ionic liquid (melting point below −10 °C) that liberates 6.9 wt.% H around 80 °C.^[^ ^434^ ^]^ In comparison, Zheng et al.’s Li(NH₃)B₃H₈ system shows good hydrogen release around 130 °C, with impurities reduced by catalysts like AlCl₃ and ZnCl₂.^[^ ^435^ ^]^

Boron and nitrogen can replace carbon in aromatic compounds, creating heterocyclic BCN systems with distinct chemical properties from their organic counterparts. Compounds such as 1,2‐BN cyclohexane and 3‐methyl‐1,2‐BN cyclopentane release hydrogen at lower temperatures (Table 9). For instance, 1,2‐BN cyclohexane releases hydrogen at 150 °C with an enthalpy of 68.5 kJ mol^−1^ H₂, in contrast to cyclohexane requiring temperatures above 350 °C. The reduced enthalpic barriers in BCN systems, such as 3‐methyl‐1,2‐BN (Δ*H*° = +32.6 kJ mol^−1^ H₂) yield efficiencies of up to 86% (efficiency = (LHVH_2_−Δ*H*)/LHVH_2_, where LHVH_2_ is the low heating value of hydrogen, and Δ*H* is the dehydrogenation enthalpy), reflecting minimal energy loss during dehydrogenation.

**Table 9:** Comparison of LOHCs to BCNs. Efficiency is calculated based on the net usable energy: LHVH_2_–Δ*H*/LHVH_2_ (LHVH_2_: low heating value of hydrogen, Δ*H*: dehydrogenation enthalpy).^[^ ^4^, ^36^, ^37^, ^63^ ^]^

	Melting Points (°C)					
Chemical	H_2_‐Rich	H_2_‐Lean	Dehydrogenation Temp. (°C)	Enthalpy (kJ mol^−1^, kJ mol^−1^ H_2_)	Efficiency	Hydrogen Capacity (wt.% H, gH_2_ L^−1^)
Cyclohexane	6	5.5	>350	205.5, 68.5	71%	7.2, 56
Methylcyclohexane	−126	−95	>350	204.9, 68.3	71%	6.2, 47.4
H18‐Dibenzyltoluene	−58	−40	>250	588.6, 65.4	73%	6.2, 56
1,2‐BN Cyclohexane	62–63	solid @ R.T.	150	205.5, 68.5	71%	7.1, 70
3‐methyl‐1,2‐BN Cyclopentane	−18	28–30	80	195.6, 32.6	86%	4.7, 42

For 3‐methyl‐1,2‐BN cyclopentane, hydrogen release took place at 80 °C using catalysts such as FeCl₂ and NiCl₂, resulting in a stable trimer of the 5‐membered ring.^[^ ^5^ ^]^ This product's viscosity makes it compatible with existing hydrocarbon infrastructure, and spent fuel was regenerated with 92% efficiency under mild conditions (Figure 12e). 1,2‐BN cyclohexane, a solid with a melting point of 75–77 °C, releases 2 equivs of hydrogen per molecule upon heating to 150 °C in toluene, yielding a trimeric product (Figure 12f).^[^ ^6^ ^]^ The activation energies for dehydrogenation are to be 78.7 kJ mol^−1^ for BN‐methylcyclopentane and 99.6 kJ mol^−1^ for 1,2‐BN cyclohexane. B─H─B bridging interactions aid these reactions by stabilizing intermediates and enabling reversible hydrogen release (Table 10).^[^ ^436^ ^]^

**Table 10:** Physical and thermodynamic properties of the common B─H bond‐based hydrogen carriers under standard conditions (* limited to 13.8 wt.% H due to LiH formation).^[^ ^5^, ^6^, ^35^, ^39^, ^377^, ^389^, ^390^, ^391^, ^392^, ^425^, ^426^, ^427^, ^428^, ^436^ ^]^

	Melting Points (°C)		Boiling Points (°C)					
Chemical	H_2_‐Rich	H_2_‐Lean	H_2_‐Rich	H_2_‐Lean	Bond Length (Å)	BDE (kJ mol^−1^)	Enthalpy (kJ mol^−1^, kJ mol^−1^ H_2_)	Hydrogen Capacity (wt.% H, gH_2_ L^−1^)
NaBH_4_	400	743	500	1575	1.16	–	180– 216, 90 – 108	10.6, –
Mg(BH_4_)_2_	400	743	500	1575	1.17 – 1.28	–	78 – 114, 39 – 57	14.9, 145–147
LiBH_4_	400	743	500	1575	1.16	–	112– 150, 56–75	18.5(13.8)*, –
1,2‐BN Cyclohexane	62–63	solid @ R.T.	–	–	1.15	–	205.5, 68.5	7.1, 70
3‐methyl‐1,2‐BN Cyclopentane	−18	28–30	–	–	0.99	–	195.6, 32.6	4.7, 42
Ammonia borane	104	–	–	–	1.08 – 1.13	419 ± 10	−21.7, –	19.6, 151
Ethylene diamine bisborane	8	–	116	–	1.08 – 1.13	–	–	9.4, –

A new BCN compound, 1,6;2,3‐bis‐BN cyclohexane, shows enhanced hydrogen storage potential. It has a hydrogen capacity of 9.4 wt.% H, nearly double that of 1,2‐BN cyclohexane (Figure 12g).^[^ ^4^ ^]^ The C─C─B─N─N─B configuration results in a free energy of 180 kJ mol^−1^ higher than its 1,2‐isomer, leading to improved reactivity but lower thermal stability of the 1,6;2,3 configuration. This reactivity complicates storage outside inert conditions.

### 5.2 Catalysts: Established and Emerging Choices

The activation of the B─H bond has been widely studied, and various catalysts, such as Fe, Mo, Ir, Rh, Ni, Pd, and Ru, have been found to be effective.^[^ ^437^, ^438^, ^439^, ^440^, ^441^, ^442^, ^443^, ^444^, ^445^, ^446^, ^447^ ^]^ By analyzing the hydrolysis mechanism of ammonia borane, Guan et al. highlighted catalytic strategies for efficient hydrogen production.^[^ ^448^ ^]^ These mechanisms are influenced by the choice of catalyst and reaction environment. Oxidative‐addition/reductive‐elimination with noble metal catalysts like Pt or Ru enables efficient bond cleavage, exemplified by Pt/C delivering rapid hydrogen release (∼8.9 wt.%) within 2 min from ammonia borane. SN2‐like pathways rely on hydroxide ions from water attacking B─H bonds to displace hydrogen. Kinetic isotope effect studies suggest that O─H cleavage is the rate‐determining step, with activation energies around 33–44 kJ mol^−1^, depending on the catalyst.^[^ ^448^, ^449^ ^]^ Highlighting a few catalysts’ performance, Ru/TiO₂ delivered a TOF of 604 min⁻¹ and Ni₀.₇Co₁.₃P on graphene oxide showed a TOF of 154 min⁻¹. Co‐CoOx/TiO₂ achieved hydrogen yields of 5905 mL min⁻¹ g_Co_⁻¹, maintaining 85% activity after five cycles.^[^ ^448^ ^]^ Iron‐based catalysts are notable for sustainability, with Fe nanoparticles hydrolyzing AB in 8 min and sustaining activity across 20 cycles, yielding 1 to 3 equivs of hydrogen, or up to 4.6 wt.% H.^[^ ^450^, ^451^ ^]^

BCNs have low dehydrogenation enthalpies, allowing efficient hydrogen release with affordable catalysts. For instance, 3‐methyl‐1,2‐BN cyclopentane has an enthalpy of 32.6 kJ mol^−1^ H₂, releasing hydrogen within 20 min at 80 °C without solvents.^[^ ^5^ ^]^ Halide complexes such as NiBr₂ and CuBr enabled 76% conversion in 5 min and complete conversion in 30 min, with bromides being the most reactive. Pd/C and Ru catalysts also show high efficiency, achieving 95% conversion at 65 °C for 1,2;4,5‐bis‐BN‐cyclohexane.^[^ ^3^ ^]^ In 1,6;2,3‐bis‐BN‐cyclohexane, Pt/C in THF released 1.47 equivs of H₂ at room temperature. This demonstrates the versatility of BCN materials and the practical use of transition metal salts for efficient hydrogen release.^[^ ^4^ ^]^

Borohydrides show promising dehydrogenation performance, particularly with effective catalysts. Table 11 compares the thermal (de)hydrogenation behaviors of borohydrides with various catalysts. For LiBH_4_, Ni on activated carbon enabled the release of 8.9 wt.% H below 300 °C due to a decreased dehydrogenation enthalpy. Fe_3_O_4_ on reduced graphene oxide decreased the dehydrogenation temperatures to as low as 74 °C.^[^ ^452^ ^]^ Cobalt‐based catalysts demonstrated impressive activity, particularly cobalt doped by boron (Co‐B).^[^ ^453^ ^]^ Liu et al. found a highly active Co‐B catalyst formed by introducing CoCl_2_ into a NaBH_4_‐NaOH solution, achieving a hydrogen generation rate of 26 L min^−1^ g^−1^ at 30 °C in a 15 wt.% NaBH_4_–5 wt.% NaOH solution.^[^ ^454^ ^]^ Liang et al. developed a porous Fe─Co─B/Ni catalyst with an activation energy of 27 kJ mol^−1^ and achieved a hydrogen generation rate of 22 L min^−1^ g^−1^ (Fe─Co─B) in a 15 wt.% NaBH_4_–5 wt.% NaOH solution.^[^ ^455^ ^]^ Additionally, Wang and colleagues have explored catalysts for NaBH_4_ methanolysis.^[^ ^456^ ^]^

**Table 11:** Hydrogen storage properties of light‐metal borohydrides modified with different catalyst.

Chemical	Catalyst	Nonisothermal Dehydrogenation *T*_onset_/ *T*_peak_ (°C)	Capacity (wt.% H)	Isothermal Dehydrogenation *T* (°C)/ time	Capacity (wt.% H)	Isothermal Hydrogenation *T* (°C)/ time	Available Capacity (wt.% H)	DeH_2_ *E*_a_ (kJ mol^−1^)	Ref.
LiBH_4_	mulberry‐like CoB	170/367	10.4	200/3 h	4.8				[457]
	Graphene	−/440	7.01	425/6 h	7.37	440/10 h	7.4		[458]
	Ti_3_C_2_ MXene	120/−	6.7	350/1 h	5.37			70.27	[459]
	h‐BN	180/435		400/2 h	12.6	400/10 h	∼7.0	155.8	[460]
	CaF_2_	118/−	9.3	450/24 h	7–8	450/24 h	8–9		[461]
	AC‐CeF_3_	180/320	13.5	−/500 s	11.8			108	[462]
	FG	148.1/−	8.2						[463]
	SrF_2_	140/−	7.6						[464]
	Ni‐AC	243/278	8.9	428/2 h	∼8			88	[465]
	TiO	240/340	10.4	350/20 min	8.2	500/100 min		114.6	[466]
	Ti_2_C_3_ Mxene	172.6/−		380/1 h	9.6	300/−		94.44 (1)	[467]
								98.27 (2)	[468]
	SiO_2_ + TiF_3_	70/483	8.3	673 K	>5.5	500	4	52.8 (1)	[469]
				3000 s		14 000 s		155 (2)	[470]
	Ce_2_S_3_	250/−	4	400/3000 s	4	400/−	4	157.82	[471]
	Fe_3_O_4_@rGO	74/−	8.88	400/60 min	3.84	400/60 min	5.45	102.02	[452]
	pyrolysis polyaniline	75/−	∼8.2	450/500 s	3.9	400/50 min	5		[472]
	Pt‐G	230/−	13.7						[473]
	Pd‐G‐60	295/−	14.9	550/5 h		400/10 h			[474]
	Li_3_BO_3_ + NbH	190/340	8.2	400/60 min		500/20 min		127.4	[475]
Mg(BH_4_)_2_	CNTs	76/117	3.76	240/10 h	5.73	350/10 h	2.5	23.98	[476]
	NbF_5_	120/−	10.04						[476]
	K_2_NbF_7_	118/−	11.3	280/200 min	6.6				[477]
	K_2_TiF_6_	105/−	11.6	280/200 min	6.4				[478]
	VF_4_@Ti_3_C_2_	91/−	10.4	275/300 min	8.2	275/300		172.0 (1)	[478]
								172.9 (2)	[478]
	Ti_3_C_2_ Mxene	149/−	12.5	260/360 min	8.7			72.91	[479]
	Ti_2_C Mxene	132/−	13.2	260/360 min	10.2			41.2	[479]
	Nb_2_C Mxene	141/−	10.1	260/360 min	7.9			106.17	[480]
NaBH_4_	LaF_3_	160/−	3.53	386/44 min	2.8	420/−	3.46	220.1	[481]
	LaH_2_	330/−	3.03	466/180 min	2.12	420/−	3.33	247	[482]
	GdF_3_	112/−	3.5	375/−	3.38	400/1.5 h	3.5	63.1	[483]
	HoF_3_	86/−	3.43	430/2 h	2.38			153	[484]
	MgFe_2_O_4_	323/−	8.4	470/60 min	6.2	420/60	45	187	[457]
Ca(BH_4_)_2_	MgF_2_	390/−	3.7	330/4 h	4.3	330/−			[458]
	NbF_5_		8.3				5		[459]

### 5.3 Summary and Conclusion for B─H Bonds

B─H bonds are promising for hydrogen storage due to their high hydrogen capacity per unit mass and per unit volume. Hydrogen release is typically carried out via thermal decomposition or hydrolysis/alcoholysis. For thermal‐driven hydrogen evolution, borohydrides typically require high temperatures. During hydrolysis and alcoholysis, B─H bonds are converted to B─O bonds at mild temperatures. Amine boranes utilize N─H^σ+^··· H^σ⁻^─B dihydrogen bonds for dehydrogenation, which normally takes place under mild conditions. Among all the borohydrides studied, NaBH₄ is particularly noteworthy for its favorable properties and low toxicity as a hydrogen carrier through hydrolysis.

There are several key challenges for this system. Certain borohydrides can be regenerated but requires extremely high pressure and/or temperatures, reducing the net energy output. The regeneration of amine boranes typically requires two steps which complicates the process. Recent work on regenerating EDAB via a one‐pot reaction shows promise, but more improvement is needed for practical applications. Nanostructured borohydrides, nanocatalysts, and reactive composites have shown dramatically enhanced properties. In addition to the known compounds, new molecules should be synthesized, particularly the BCN systems.

## 6 Summary and Outlook

Till now no single hydrogen supply pathway is ideal; each has its own advantages and disadvantages. Significant research has concentrated on hydrogen carriers that contain B─H, C─H, N─H, or O─H bonds. Several factors such as BDE, reaction pathways, and catalysts are crucial in hydrogen cycling performance. Additionally, safety, infrastructure, and economics associated with these carriers' production, transport, and use also play important roles in practical applications.

BDE serves as a useful parameter for initially evaluating or predicting the dehydrogenation characteristics of potential carrier compounds. It measures the energy required to break a specific bond (X─H), providing insights into bond stability and hydrogen release feasibility. Although it does not dictate all reaction dynamics, BDE can be useful when categorizing hydrogen carriers. When comparing hydrogen storage performance, the differences in decomposition temperatures can result from factors such as reaction pathways, catalyst, and decomposition products. For example, the BDE of B─H bonds in ammonia borane is 419 ± 10 kJ mol^−1^, while the C─H bonds in ethane have a BDE of about 410 kJ mol^−1^. However, ethane requires high temperatures (around 500 °C) for noncatalytic dehydrogenation, in contrast to 100 °C required for AB to decompose noncatalytically. The difference is mainly due to the extensive dihydrogen bond interactions within AB.

**Figure 14:**
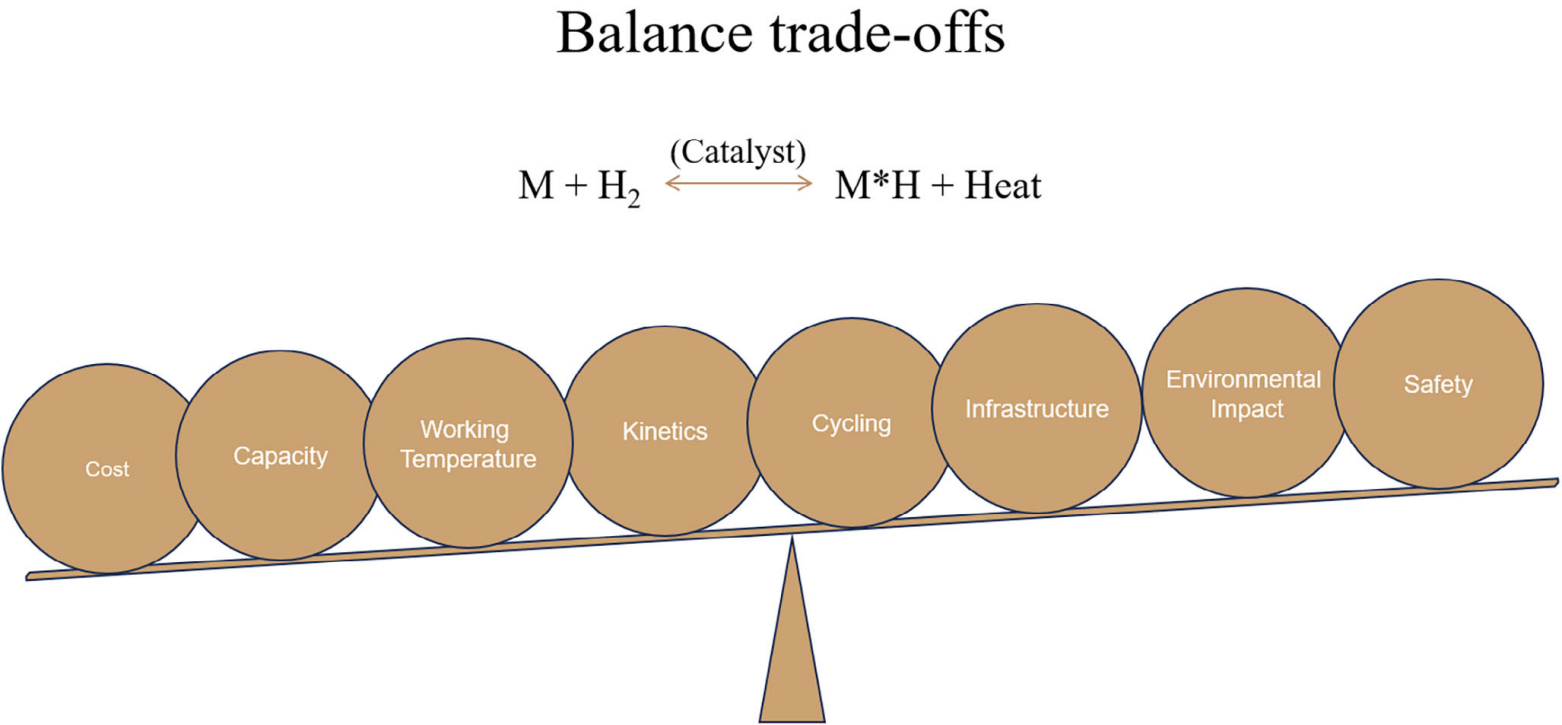
The see‐saw of parameters to balance when considering hydrogen carriers (M = host hydrogen storage materials).

Years of research have advanced hydrogen technology for potential applications in sectors such as emergency, shipping, and mobility. Efforts are underway to move some technologies from laboratory experiments to pilot‐scale demonstrations, aiming for industrial‐scale operations. Designing an effective hydrogen carrier system requires balancing several parameters; overemphasizing one aspect can compromise the whole system (Figure 14). For example, certain additives can improve reaction kinetics, but they reduce the hydrogen density, and can induce impurities, and complicate the regeneration step. This highlights the complexity and balancing act involved in developing hydrogen carriers.

Liquid‐state carriers such as LOHCs provide streamlined closed‐loop systems with more straightforward engineering than other carriers. They integrate easily into existing infrastructure, such as pipelines and refineries, allowing for quick deployment in a large scale. However, challenges remain such as high C─H bond dissociation energies and expensive catalysts, and more ongoing research is needed to improve efficiency and stability. Formic acid and methanol are efficient O─H carriers, ideal for easy storage and transport. The latest electrochemical production and reforming offer promising opportunities. Converting CO_2_ into these compounds has gained interest since it reduced carbon footprints.

Ammonia is a promising hydrogen carrier due to its high hydrogen content and established transport infrastructure. However, its production and hydrogen generation via thermal cracking are energy intensive. New ammonia synthesis via electrochemical reduction of nitrogen holds promise to reduce the energy cost. Novel electrocatalytic decomposition can improve the efficiencies in ammonia cracking. Further improvement requires not only effective catalysts, but also efficient reactors and accessories. Despite high decomposition temperatures, borohydrides and their composites are promising for hydrogen storage due to their stability and high gravimetric density. Research focuses on lowering (de)hydrogenation temperatures and exploring new hydride combinations. BCNs feature low‐temperature hydrogen release but struggle with regeneration efficiency, reflecting the challenges in optimizing B─H‐based hydrogen carriers for the hydrogen economy.

Transition to a green hydrogen economy involves complex decisions regarding hydrogen carriers, requiring consideration of key factors such as thermodynamics, kinetics, costs, environmental impact, and safety. Each carrier has advantages and challenges that must be addressed for practical use. Future research includes but is not limited to discovering new hydrogen‐rich molecules, developing cost‐effective catalysts, exploring alternative production methods, and determining the best applications. Considering the various parameters affecting the applications (Figure 14), effective collaboration between research institution and industry is essential to optimize these systems and realize their potential for large‐scale hydrogen applications.

## Conflict of Interests

The authors declare no conflict of interest.

## Data Availability

Data sharing is not applicable to this article as no new data were created or analyzed in this study.
